# Eating cognitions, emotions and behaviour under treatment with second generation antipsychotics: A systematic review and meta-analysis

**DOI:** 10.1016/j.jpsychires.2023.02.006

**Published:** 2023-04

**Authors:** Hiba Mutwalli, Johanna Louise Keeler, Sevgi Bektas, Namrata Dhopatkar, Janet Treasure, Hubertus Himmerich

**Affiliations:** a Section of Eating Disorders, Department of Psychological Medicine, Institute of Psychiatry, Psychology & Neuroscience, King's College London, London, UK; b Department of Clinical Nutrition, College of Applied Medical Sciences, Imam Abdulrahman Bin Faisal University, Dammam, Saudi Arabia; c Department of Psychology, Hacettepe University, Ankara, Turkey; d Eating Disorders Unit, Bethlem Royal Hospital, South London and Maudsley NHS Foundation Trust (SLaM), London, UK

**Keywords:** Eating behaviour, Appetite, Food craving, Food addiction, Atypical antipsychotic, Second generation antipsychotic

## Abstract

Weight gain and metabolic disturbances are frequent in people treated with second generation antipsychotics (SGA). We aimed to investigate the effect of SGAs on eating behaviors, cognitions and emotions, as a possible contributor to this adverse effect. A systematic review and a meta-analysis were conducted following the Preferred Reporting Items for Systematic reviews and Meta-Analyses (PRISMA) guidelines. Original articles measuring outcomes relating to eating cognitions, behaviours and emotions, during treatment with SGAs were included in this review. A total of 92 papers with 11,274 participants were included from three scientific databases (PubMed, Web of Science and PsycInfo). Results were synthesized descriptively except for the continuous data where meta-analyses were performed and for the binary data where odds ratios were calculated. Hunger was increased in participants treated with SGAs with an odds ratio for appetite increase of 1.51 (95% CI [1.04, 1.97]; z = 6.40; *p* < 0.001). Compared to controls, our results showed that craving for fat and carbohydrates are the highest among other craving subscales. There was a small increase in dietary disinhibition (SMD = 0.40) and restrained eating (SMD = 0.43) in participants treated with SGAs compared to controls and substantial heterogeneity across studies reporting these eating traits. There were few studies examining other eating-related outcomes such as food addiction, satiety, fullness, caloric intake and dietary quality and habits. Understanding the mechanisms associated with appetite and eating-related psychopathology changes in patients treated with antipsychotics is needed to reliably inform the development of effective preventative strategies.

## 1 Introduction

Antipsychotics are classified into first-generation antipsychotics (FGAs), also known as typical antipsychotics and second-generation antipsychotics (SGAs), the so called atypical antipsychotics. SGAs are the first line of treatment for patients with schizophrenia and are also frequently used as mood stabilizers (Cepaityte et al., 2021; Lieberman, 2004). Whereas FGAs are known to cause extrapyramidal side effects such as akathisia and parkinsonism, whereas SGAs have been reported to cause metabolic side effects including an increase in appetite, weight gain and obesity (Deng, 2013).

Clozapine is regarded as the first SGA, introduced in the 1970s (de Maio, 1972). Other SGAs such as olanzapine were introduced in the 1990s and labelled as “atypical antipsychotics” (Moore et al., 1992). Some SGAs such as clozapine and olanzapine were found to induce a significant amount of weight gain (Alonso-Pedrero et al., 2019; Himmerich et al., 2015). Antipsychotic-related weight gain is thought to be associated with their affinity to histamine H1, dopamine and serotonin receptors (Kim et al., 2007; Kroeze et al., 2003; Roerig et al., 2011).

SGAs differ regarding their effect on body weight. Clozapine and olanzapine are associated with the most weight gain compared to other SGAs (Dayabandara et al., 2017). Quetiapine and risperidone lead to moderate weight gain and amisulpiride, aripiprazole, asenapine, lurasidone and ziprasidone are reported to be weight-neutral in most patients (Alonso-Pedrero et al., 2019; Barton et al., 2020; Dayabandara et al., 2017; Himmerich et al., 2015; Pillay et al., 2018; Ribeiro et al., 2018; Rognoni et al., 2021; Zhao et al., 2016). Additionally, there are individual differences in weight change between different patients who take the same medication. For example, in a study published by Kinon et al. (2001), some patients lost weight, gained no weight, or gained more than 20 kg during 3 years of treatment with olanzapine.

Changes in eating behaviours occur in a wide range of psychiatric disorders (Milaneschi et al., 2017; Wen Chi et al., 2015). For example, patients with acute schizophrenia, depression or anorexia nervosa might show more restrictive eating behaviours during acute episodes and tend to lose weight (Garfinkel et al., 1983), whereas patients with atypical depression or dementia can experience hyperphagia (Hsiao et al., 2013). Dementia was also shown to be related to the development of pica (Wen Chi et al., 2015). When patients recover from acute psychiatric disorders, this recovery is often associated with a normalisation of their eating behaviour and a return to their usual body weight. This phenomenon had already been described in the pre-psychopharmacological era by Kraepelin (1904) and Kryspin-Exner (1947).

Many studies have examined weight gain and weight-related outcomes during treatment with antipsychotics whereas fewer studies have specifically examined eating-related outcomes including eating behaviours, cognitions, emotions and the regulation of appetite. Understanding the mechanism of weight gain may be of value in devising treatment methods to counteract this unwelcome side effect. These could be psychological treatments or pharmacological approaches addressing changes in appetite or food-related behaviour and emotions.

## 2 Materials and methods

**Fig. 1:**
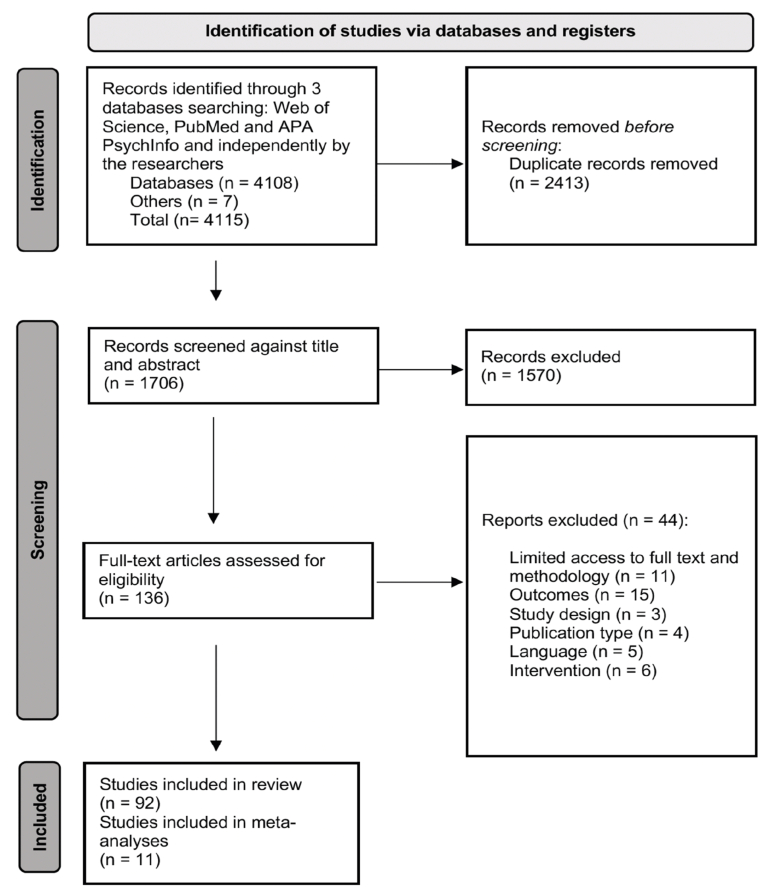
PRISMA flowchart illustrating the identification of included studies.

This systematic review and meta-analysis followed the Preferred Reporting Items for Systematic reviews and Meta-Analysis (PRISMA) guidelines (Page et al., 2021). See S.1 for the PRISMA 2020 main checklist, S.2 for PRISMA 2020 abstracts checklist and Fig. 1 for PRISMA identification of studies flow chart. A systematic search for eligible publications was conducted between the database date of inception until November 1st, 2021, using three databases: Web of Science, PubMed and APA PsycInfo (Ovid).

The search was conducted using the following terms: Eating behaviour, binge eating, food craving, carbohydrate craving, food intake, dietary intake, food preference, appetite, hunger, food addiction, junk food, food, food choice, eating habits, motivation to eat, food responsiveness, overeating, hyperphagia, energy intake, caloric intake, desire to eat, polyphagia, pica, antipsychotic, amisulpride, aripiprazole, asenapine, clozapine, lurasidone, olanzapine, paliperidone, quetiapine, risperidone, cariprazine, brexpiprazole, clotiapine, iloperidone, molindone and ziprasidone. See S.3 for further details on the search strategy.

Additional publications were identified through a manual hand search through reference lists of relevant papers. A protocol and search strategy were developed by H.H. and H.M., which was prospectively registered with the Open Science Framework (protocol accessible at: https://osf.io/6esm8/).

### 2.1 Inclusion and exclusion criteria

Original articles of clinical studies published at any date were included in our review. Case reports, review articles, letters and animal studies were excluded. Only studies written in English were considered in our systematic review. Studies were included if they measured outcomes relating to eating behaviour, food intake, and the regulation of appetite in patients or healthy participants who were exposed to SGAs. The duration of exposure to the SGA was unrestricted. Studies in which the effect of SGAs on weight change was the only measured outcome were also excluded. Objective and subjective outcome measures were accepted in our review. Studies scoring less than 50% in the quality assessment – which indicates a high risk of bias - were excluded. For more details about the inclusion and exclusion criteria see S.4.

### 2.2 Study selection

All publications identified by the search were screened independently by two reviewers (H.M. and S.B.). Endnote and Rayyan, an online software for systematic reviews (Ouzzani et al., 2016) were used for the management of the search results. After the removal of duplications of the results, titles and abstracts were screened against the aforementioned eligibility criteria. Queries regarding eligibility were discussed with the wider research team (J.T., H.H. and J.K.) before a decision on inclusion/exclusion was reached. Authors were contacted to provide missing full-texts and/or information where needed.

### 2.3 Data extraction

Data extraction was performed by H.M. and reviewed by S.B. Extracted data included the titles, abstracts, authors and lead author name and contact details, origin and aim of the study, recruitment methods and setting, study design, duration of the study/intervention, funding and possible conflict of interest, inclusion and exclusion criteria, sample size and population characteristics, diagnosis and criteria of diagnosis, intervention/s, comparisons, drug dosage, outcomes and measures, and the main findings. All data were collated in a Microsoft Excel spreadsheet.

### 2.4 Quality appraisal

Two reviewers (H.M. and S.B.) conducted a quality appraisal using the Joanna Briggs Institute critical appraisal tool (Moola et al., 2020). This tool offers different checklists for different study designs: cross sectional, case control, cohort, diagnostic, quasi-experimental and randomized controlled trials (RCTs), facilitating the comparison of quality across study types. The quality appraisal focused on the methodological quality of publications, in order to examine the possibility of bias in the study design, methods and results. The available JBI appraisal tools do not offer versions designed for open-label trials. Therefore, we used the quasi-experiment version to appraise open-label trials. Overall, the quality assessment decision to include or exclude studies was based on the frequency of responses with “no” or “unclear” with a 50% or more indicating high risk of bias and thus the exclusion of a report. The detailed quality appraisal of candidate studies is shown in S.5.

### 2.5 Quantitative analysis

For continuous outcomes such as eating behaviour (restraint, disinhibition) and hunger, meta-analyses were performed where sufficient (two or more) studies were available. Odds ratios were calculated for binary outcomes such as appetite increase. Both the meta-analyses and odds ratios utilized random effects models, using the DerSimonian and Laird method (DerSimonian and Laird, 1986) which was used to calculate standardized mean of differences (SMD) or odds ratios. All quantitative analyses were conducted using the meta set and summarize commands in Stata 16 (StataCorp, 2019).

Where possible, effect sizes (Cohen's *d*) were reported for studies that were not included in the meta-analysis, which were calculated using means and standard deviations reported in the studies. Effect size values were considered small (*d* = 0.2), moderate (*d* = 0.5) or large (*d* = 0.8) (Cohen, 2013).

### 2.6 Sensitivity analyses

Sensitivity analyses and heterogeneity between studies were assessed using the Higgins I^2^ function which was considered high when the I^2^ was higher than 75%. The Egger's test was also used together with funnel plots to identify any potential publication bias. The Duval and Tweedie trim and fill method (Duval and Tweedie, 2000) was used to identify present or absent studies causing funnel plot asymmetry, and to adjust for these studies.

### 2.7 Qualitative data synthesis

All studies that met the criteria for investigating eating cognitions, emotions and behaviours, but were not included in the meta-analyses and odds ration calculations, were reviewed and descriptively reported in this review, including available data from interventional and observational studies.

## 3 Results

### 3.1 Characteristics of included studies and participants

A total of 92 studies with a total of 11,274 participants were eligible for inclusion in this systematic review. Of those, 11 studies were included in the quantitative analyses. Articles were published from the following countries: United States (n = 35; 37.6%), Italy and Australia (n = 6; 6.4% each); Canada and India (n = 5; 5.3% each); Germany, Turkey and Iran (n = 4; 4.3% each); Japan (n = 3; 3.2%); Netherlands, Israel, Spain, France, Hungary, UK, Thailand and Denmark (n = 2; 2.1% each); Austria, Taiwan, South Korea, Switzerland and Brazil (n = 1; 1% each). For more details about the summary of included studies see Table 1.

**Table 1:** Summary of included studies Interventional studies.

Author (year)	Sample size	Sample characteristics	Age (years)^a^	Study design	Agent/comparison^b^	Duration^c^	Outcome	Instrument	Findings
Aman et al. (2005)	101 (49 Risperidone, 52 placebo)	Autism disorder	8.8 ± 2.7	RCT	2.5–3.5 mg/day risperidone/placebo	6 months	Appetite	Reported	Baseline appetite increase: - 12% risperidone. -11.5% placebo. 8 weeks appetite increase: -Moderate/severe: 32.6% risperidone and 9.6% placebo (d: 0.81). -Mild: 49% risperidone and 29% placebo (d: 0.81).
Ballon et al. (2018)	24 (7 olanzapine, 7 Ileoperidone, 10 placebo)	Healthy	18–35	RCT	Target dose 5 mg/d olanzapine & 6 mg/d ileoperidone/placebo	4 weeks	Dietary composition, caloric intake & hunger	Dietary composition & caloric intake calculated from meals consumed at experimental sessions, VAS for hunger	Dietary composition in the olanzapine group -Increase in carbohydrates consumption (+22 ± 11g, *p* = 0.09). -Increase in protein consumption (+14 ± 11g, *p* = 0.23). -Increase in fat consumption (+13 ± 7 g, *p* = 0.09). Caloric intake in the olanzapine group -25% increase in days 14 and 28 (+268 ± 77 kcal, *p* = 0.01). Hunger in the olanzapine group -Increase in day 28 (d: 0.489). Hunger in the placebo group -Increase in day 28 (d: 0.75).
Black et al. (2014)	111	Borderline personality disorder	18–45	RCT	150–300 mg/d quetiapine/placebo	8 weeks	Appetite	Reported	Appetite increase (# 26, 27%), of which 25 were related to the study drug. -Placebo (# 4). -Low-dose quetiapine (# 9). -Moderate-dose quetiapine (# 13). -Appetite increase seen more frequently in participants taking higher dosage of quetiapine (HR: 3.89)
Daurignac et al. (2015)	19 (13 olanzapine, 6 placebo)	Healthy	18–35	RCT	5 then 10 mg/d olanzapine/placebo	2 weeks	- Caloric intake -Dietary composition	24-h dietary recall & experimental meals	Caloric intake -No change in caloric intake during experimental breakfast. Dietary composition -Increase in protein intake in the olanzapine comparing to the placebo group (d: 1.36).
Findling et al. (2015)	403	Bipolar I disorder, manic or mixed episode	13.8 ± 2.0	RCT	2.5, 5 & 10 mg/d asenapine/placebo	3 weeks	Appetite	Reported	Twice the number of participants reporting appetite increase seen in the asenapine group
Fountaine et al. (2010)	30	Healthy	18–49	RCT	5 mg/d then 10 mg/d olanzapine/placebo	2 weeks	Caloric intake	Caloric content measured in all meals and snacks	Caloric intake: -Higher in the olanzapine group than placebo. -18% increase from baseline to week 2 in the olanzapine group.
Ghanizadeh (2016)	36	Tic disorder	6–18	RCT	3.8–4.5 mg/d twice weekly aripiprazole/3.5–4 mg/d daily aripiprazole	8 weeks	Appetite	Checklist of ADRS developed by Ghanizadeh and Haghighi (2014)	-Appetite increase is the most reported ADRS. --Appetite increase in 6 (31.6%) of the daily and 2 (10.3%) of the twice weekly aripiprazole groups (d: 0.15). --The odds ratio for appetite increase is 3 (95% CI: 0.50 to 17.70).
Guardia et al. (2004)	60 (29 Olanzapine, 31 Placebo)	Alcohol-dependence disorder	18–60	RCT	7.54 mg/d olanzapine/placebo	12 weeks	Appetite	Reported	Appetite increase in 25% of the olanzapine and 10.3% of the placebo group (d: 0.35).
Hellings et al. (2006)	40	Mental retardation & autism spectrum disorder	22 ± 13.1	RCT	0.05 mg/kg/dayrisperidone/placebo	46 weeks	Appetite	Reported	-Increase in appetite in week 4–6. -Most reported drug adverse effect.
Kane et al. (2001)	71 (37 clozapine, 34 haloperidol)	Schizophrenia & schizoaffective disorder	41 ± 10	RCT	141 mg/d clozapine/5 mg/d haloperidol	29 weeks	Appetite	Reported	Decrease in appetite in the haloperidol when compared to the clozapine group (d: 0.76).
Karagianis et al. (2009)	149 (84 ODO, 65 SOT)	Schizophrenia, schizoaffective disorder, BD & other psychotic disorders	39 ± 13	RCT	13.87 mg/d ODO, 13.23 mg/d SOT/placebo	16 weeks	Appetite	VAS	-Change in appetite did not differ between groups after 16 weeks of treatment. -Appetite decrease in the ODO group when compared to the SOT (d: 0.37). -**Increased appetite:** ODO (# 9, 10.7%)**,** SOT (# 10, 15.4%) and total (# 19, 12.8%). -**Decreased appetite:** ODO (# 3, 3.6%)**,** SOT (# 0, 0.0%) and Total (# 3, 2.0%).
Kent et al. (2013b)	77 (25 low dose risperidone, 25 high dose risperidone, 27 placebo)	Autism disorder	9 ± 3.1	RCT	20–45 kg: 0.125–1.25 mg/d, >45 kg: 0.175–1.75 mg/d risperidone/placebo	6 weeks	Appetite	Reported	Appetite increase -The most reported ADRS. -Low dose risperidone (17%). -High dose risperidone (35%). -Overall (26%).
Lindsay et al. (2006)	20	Autism disorder	8.38 ± 2.21	RCT	1.8 mg/day risperidone/placebo	8 weeks	Caloric intake & dietary composition	FFQ	-Caloric intake 120% higher than RDA in 8 participants. -Lower caloric intake in 2 participants (49% and 37% lower than RDA). -Mean intakes of carbohydrates, proteins and fats were higher than the recommended DRI. -Most of the participants had an adequate intake of protein (# 20), carbohydrates (# 18) and fat (# 19). -Significant lower carbohydrates intake in 2 participants (87% and 55% lower than RDA).
Litten et al. (2012)	218, (113 placebo 105 quetiapine)	Alcohol-dependence disorder	18–64	RCT	Target dose of 400 mg/d quetiapine/placebo	17 weeks	Appetite	Reported	Increased appetite in the treatment group (# 12, 11%) and placebo group (# 1, 1%).
McCracken et al. (2002)	101, (49 risperidone, 51 placebo)	Autism disorder	8.8 ± 2.7	RCT	1.8 mg/d risperidone/placebo	24 weeks	Appetite	Reported	Mild appetite increase: -Risperidone group: # 24, 49%. -Risperidone vs. placebo: d: 0.44 Moderate appetite increase: -Risperidone group: # 12, 24%. -Risperidone vs. placebo: d: 0.53. Appetite decrease: -Risperidone group: # 3, 6%. -Risperidone vs. placebo: d: 0.06.
Nagaraj et al. (2006)	40	Autism disorder	2–9	RCT	0.5 mg/day then 1 mg/d risperidone/placebo	6 months	-Appetite	Reported	Appetite increase in the risperidone group (# 17, 42.5%).
Navari et al. (2020)	30 (15 olanzapine, 15 placebo)	Cancer	39–79	RCT	5 mg/d olanzapine/placebo	7 days	Appetite	NRS	Baseline to day 7 appetite increase in the treatment group compared to placebo (d: 1.34).
Razjouyan et al. (2018)	48 (25 risperidone, 23 aripiprazole)	ADHD	3–6	RCT	Initial dose 0.25 mg/d, maximum dose 1 mg/d risperidone & initial dose 1.25 mg/d maximum dose 5 mg/d aripiprazole/placebo	12 weeks	Appetite	Reported	-Appetite increase in the aripiprazole group (# 4, 20%). -Appetite increase in the risperidone group (# 1, 5%).
Roerig et al. (2005)	48 (16 olanzapine, 16 Risperidone/16 placebo)	Healthy	18–60	RCT	8.75 mg/d olanzapine & 2.875 mg/d risperidone/placebo	2 weeks	caloric intake, appetite and hunger	-**Caloric intake** Monitoring and calculating food intake-**Appetite, hunger** Computerized rating analog	-Higher caloric intake at dinner session in the olanzapine group than placebo and risperidone After 1 week: -Risperidone vs placebo (d: 0.3341). -Olanzapine vs placebo (d: 0.5067). -Risperidone vs olanzapine (d: 0.7729). After 2 weeks: -Risperidone vs placebo (d: 0.5457). -Olanzapine vs placebo (d: 0.1892). -Risperidone vs olanzapine (d: 0.7359). -Appetite increase in the olanzapine **(#** 6, 37.5%), risperidone (# 7, 43.8%) and placebo (# 4, 25%). -No difference in hunger in all groups. -Hunger ratings were numerically greater than baseline.
Snyder et al. (2002)	110 (53 Risperidone, 57 placebo)	Conduct and disruptive behaviour disorder	5–12	RCT	0.98 mg/day risperidone/placebo	7 weeks	Appetite	Reported	-Appetite increase in the risperidone (# 8, 15.1%) compared to the placebo group (# 2, 3.5%). -Appetite decrease in the risperidone (# 4, 7.5%) compared to the placebo group (# 2, 3.5%).
Srivastava et al. (2012)	50 (25 olanzapine & 25 placebo)	BD	40.8 ± 11.5	RCT	Olanzapine/placebo	1 week	Appetite	Reported	-Higher appetite increase reported in the olanzapine (# 24, 54.2%) compared to the placebo (# 22, 22.7%) groups (d: 0.65). -Number needed to harm for appetite increase: 4 (95% CI = 1–21).
Teff et al. (2013)	30	Non-specified psychiatric disorders	Olanzapine 26.1 ± 3.5, aripiprazole 25.9 ± 4.3, placebo 29.9 ± 7.5	RCT	10 mg/d olanzapine & 10 mg/d aripiprazole/placebo	12 days	Hunger and caloric intake	**Caloric intake** weighed food items prior to and after each experimental meal **Hunger** VAS	No change in caloric intake and hunger in all groups.
Tohen et al. (2002)	251 (125 olanzapine, 126 divalproex)	Bipolar I disorder, manic or mixed episode	18–75	RCT	17.4 mg/day olanzapine/1401.2 mg/day divalproex	3 weeks	Appetite	Reported	Appetite increase in the olanzapine (# 15, 12%) and divalproex (# 3, 2.4%) groups (d: 0.38).
Tohen et al. (2003)	251 (125 olanzapine, 126 divalproex)	BD, manic or mixed episode	18–75	RCT	5–20 mg/day olanzapine/500–2500 mg/day divalproex	47 weeks	Appetite	Reported	Appetite increase in the olanzapine (# 17, 13.6%) and divalproex groups (# 7, 5.6%) (d: 0.25).
Tollefson et al. (1997)	1996 (Olanzapine 1,336, Haloperidol 660)	Schizophrenia, schizoaffective & schizophreniform disorders	38.7 ± 11.6	RCT	13.2 mg/d olanzapine/11.8 mg/d haloperidol	14 months	Appetite	Reported	-Appetite increase in the olanzapine (# 313, 24%) and the haloperidol group (# 79, 12.4%). -Appetite decrease in the olanzapine (# 149, 11.4%) and the haloperidol group (# 115, 18.1%).
Agarwal and Sitholey (2006)	23	Symptoms of acute and transient psychotic disorders	14 ± 1.3	Open-label	12.7 mg/d olanzapine	6 weeks	Appetite	DOTES	-Mild increase in appetite (# 12, 52%), moderate increase in appetite (# 2, 8.6%). -Mild Decrease in appetite (# 1, 4.3%).
Bitter et al. (2010)	265	Schizophrenia	18–65	Open-label	5–20 mg/d ODO & SOT	12 weeks	Appetite	**VAS**	No difference in appetite between the ODO and SOT groups.
Bobo et al. (2011)	23	Bipolar depression	18–60	Open-label	13.3 mg/d ODO & 16.5 mg/d SOT	8 weeks	Food craving and eating behaviour	FCI and TFEQ	Food craving -No change in FCI scores. Eating behaviour -No change in TFEQ scores.
Costa e Silva et al. (2001)	94	Schizophrenia, schizophreniform disorder, & schizoaffective disorder	31.8 ± 8.3	Open-label	11.4 mg/d olanzapine/12.7 mg/d haloperidol	6 weeks	Appetite	Reported	Appetite increase in the olanzapine group (# 14, 14.9%)
Findling et al. (2003)	16	Schizophrenia, schizoaffective disorder, & schizophreniform disorder	13.8 ± 1.5	Open-label	12.4 mg/d olanzapine	8 weeks	Appetite	Reported	Appetite increase (# 11, 68.8%).
Findling et al. (2011)	96	BD	6.9 ± 1.7	Open-label	6.5 mg/d aripiprazole	a range of 2.0–18.1 weeks	Appetite	Reported	Appetite increase (#38, 40%). -Responders group (# 26, 43%). -Non-responders group (# 12, 33%).
Gagliano et al. (2004)	20	Autism disorder	6.0 ± 2.4	Open-label	1.26 mg/d risperidone	24 weeks	Appetite	Reported	Appetite increase (# 12, 60%).
Ghaeli et al. (2014)	15	Autism disorder	6.5 ± 3.5	Open-label	0.06 mg/kg/d, maximum dose 3 mg/d risperidone	8 weeks	Appetite	Reported	Appetite increase (# 8, 53.3%).
Gothelf et al. (2002)	20	Schizophrenia	17 ± 1.6	Open-label	14 mg/d olanzapine/4.5 mg/d haloperidol	4 weeks	Caloric intake and dietary composition	Monitoring and calculating food intake	Caloric intake: -Increase from baseline to week 4: 27.7% (d: 0.592). Dietary composition: -No change.
Ho et al. (2014)	81	Tic disorder	8.3 ± 3.4	Open-label	2.84 mg/d aripiprazole	14 weeks	Appetite	Reported	-Appetite increase (#18, 22.2%). -Appetite decrease (#5, 6.2%).
Ishitobi et al. (2012)	23	Pervasive developmental disorder	15.1 ± 3.9	Open-label	2.8 mg/d aripiprazole	14.9 ± 8.4 weeks	Appetite	Reported	Appetite increase (#11, 47.8%).
Kemner et al. (2002)	25	Pervasive developmental disorder not otherwise specified & autistic disorder	6 ± 16	Open-label	10.7 mg/d olanzapine	12 weeks	Appetite	Reported	Appetite increase (#14, 56%).
Kent et al. (2013a)	79	Autism disorder	9 ± 3.1	Open-label	Maximum dose for 20 - <45 kg: 1.25 mg/d, >45 kg: 1.75 mg/d risperidone	26 weeks	Appetite	Reported	Appetite increase (#9, 11%).
Lyon et al. (2009)	11	Tourette's disorder	13.36 ± 3.33	Open-label	4.5 mg/d aripiprazole	10 weeks	Appetite	Reported	-Mild increase in appetite (# 7, 64%). -Decrease in appetite (# 6, 55%).
Malone et al. (2002)	22	Autism disorder	7.1 ± 3.3	Open-label	Maximum dose 6 mg/d risperidone	7 months	Appetite	Reported	Appetite increase (# 7, 31.8%).
Park et al. (2013)	20 (10 Ziprasidone, 10 Olanzapine)	Brief psychotic disorder, schizophreniform disorder, schizophrenia, or schizoaffective disorder	18–65	Open-label	109 mg/d ziprasidone & 11.6 mg/d olanzapine	12 weeks	Appetite	VAS	No change in appetite in both groups.
Troost et al. (2005)	26	Autism disorder, asperger disorder & pervasive developmental disorder not otherwise specified	5–17	Open-label	1.9 mg/d risperidone	24 weeks	Appetite	Reported	-Mild increase in appetite (# 13, 49%). -Moderate increase in appetite (# 3, 8%).
Ghanizadeh and Haghighi (2014)	60 (aripiprazole 31 & risperidone 29)	Tic disorder	Aripiprazole 11.12 ± 3.3 Risperidone 10.22 ± 2.3	Randomized trial	Maximum dose <40 kg: 10 mg/d, >40 kg: 15 mg/d aripiprazole & <40 kg: 2 mg/d, >40 kg: 3 mg/d risperidone	8 weeks	Appetite	Assessed using a checklist of drug adverse effects	-Appetite increase between the aripiprazole and risperidone groups (d: 0.04). -Appetite increase in the aripiprazole group (# 8, 25.8%). -Appetite increase in the risperidone group (# 8, 27.6%). -Decreased appetite in the aripiprazole group (# 4, 12.9%).
Jakobsen et al. (2018a)	428	Schizophrenia spectrum disorder	38.6 ± 12.4	Randomized trial	Olanzapine, clozapine & quetiapine	2 years	Dietary quality	DQS and 8-items FFQ	DQS score negatively correlated with SGA use.
Scahill et al. (2016)	97	Autism spectrum disorder	6.9 ± 2.35	Randomized trial	Maximum dose 14–20 kg: 1.75 mg/d, 20 - <45 kg: 2.5 mg/kg, >45 kg: 3.5 mg/d risperidone/risperidone and parent training	24 weeks	Appetite	Reported	-Appetite increase in the risperidone group (# 75) in the first 8 weeks of treatment. -No change in appetite after 22 weeks.
Smith et al. (2012)	30 (13 olanzapine & 17 risperidone)	Schizophrenia	41.2 ± 7.3	Randomized trial	25.2 mg/d olanzapine & 6.1 mg/d risperidone	5 months	-Appetite	VAS and EBA	-No appetite change. -rend for appetite decrease.
Mathews et al. (2012)	25	Healthy	27.5 ± 5.9	Non-randomized trial	Fixed 10 mg/d olanzapine	1 week	Eating behaviour and caloric intake	TFEQ, monitoring and calculating food intake	Eating behaviour -Higher overall score of the TFEQ 1 week after treatment (d: 0.34). -Higher dietary disinhibition subscale of the TFEQ 1 week after treatment (d: 0.32). -No difference in the dietary restraints and hunger subscales of the TFEQ. Caloric intake -25% increase in food intake. -35% increase in breakfast session food intake.
Onor et al. (2007)	135	Alzheimer's disease	72.18 ± 3.2	Non-randomized trial	1 mg/d risperidone	12 weeks	Appetite	NPI	No differences in appetite after treatment.
Kinon et al. (2005)	1191 (183 rapid weight gainers, 1008 non-rapid weight gainers)	Schizophrenia or schizoaffective disorder	Rapid weight gainers 23.9 ± 3.9, non-rapid weight gainers 26.4 ± 5.2	Data from RCT	5–20 mg/d olanzapine	52 weeks	Appetite	Reported	Appetite increase in the rapid weight gainers (44%) when compared to the non-rapid weight gainers (25%) at 6 weeks of follow up.
Kluge et al. (2007)	30 (15 Clozapine, 15 olanzapine)	Schizophrenia, schizophreniform, or schizoaffective disorder	18–65	Data from RCT	Mean modal dose 266.7 mg/d clozapine & 21.2 mg/d olanzapine	6 weeks	Food craving, appetite, caloric intake and binge eating	A standardized binary scale	Appetite increase: -Clozapine (# 14, 93.3%) -Olanzapine (# 15, 100%). Increase in caloric intake: -Clozapine (# 13, 86.67%). -Olanzapine (# 14, 93.3%). Increase in food craving: -Increase in participants with binge eating. -Higher for fatty and sweets in the olanzapine (48.9%) than the clozapine group (23.3%). Higher binge eating: -Higher in the olanzapine (16.7%) than the clozapine group (8.9%). -Noticed earlier in the olanzapine group.
Observational studies									

Author (year)	Sample size	Sample characteristics	Age in years^a^	Study design	Agent/comparison^b^	Duration^c^	Outcome	Instrument	Findings
Abbas and Liddle (2013)	60 (20 olanzapine, 20 FGAs, 20 controls)	Schizophrenia	18–65	Case-control	Olanzapine, FGAs/controls	15.1 ± 19 months	Food craving	FCI	General craving d cohen: -Olanzapine vs typical: 0.3571 (−0.9818 – 0.2676) -Olanzapine vs controls: 0.1127 (−0.5075 – 0.733) Carbohydrates craving d cohen: -Olanzapine vs typical: 0.3006 (−0.9239 – 0.3226) -Olanzapine vs controls: 0.1804 (−0.4407 – 0.8015) Sweet craving d cohen: -Olanzapine vs typical: 0.1676 (−0.7885 – 0.4533) -Olanzapine vs controls: 0.0497 (−0.6696 – 0.5702) Fat craving d cohen: -Olanzapine vs typical: 0.4935 (−1.1226 – 0.1357) -Olanzapine vs controls: 0.2376 (−0.3844 – 0.8596) Fast food fat craving d cohen: -Olanzapine vs typical: 0.5147 (−1.1446 – 0.1153) -Olanzapine vs controls: 0.1572 (−0.778 – 0.4635) -No between groups difference in food craving. -Lowest numerical craving scores seen in the control group. -Highest numerical craving scores in the FGAs treated participants.
Aman et al. (2015)	84 (57 risperidone, 27 controls)	Autism disorder	8.82 ± 2.69	Cohort	2.47 mg/d risperidone/controls	Average of 21.4 months	Appetite	Reported	Appetite increase in the risperidone (# 24, 42.1%) and controls (# 5, 22%) groups (d: 0.41).
Cicala et al. (2020)	116	Non-specified psychiatric disorders	11.4 ± 3.5	Cohort	0.25–7.5 mg/d risperidone, 2–20 mg/d aripiprazole, 2–20 mg/d olanzapine, clozapine 25–300 mg/d & 25–250 mg/d quetiapine	12 months	Overeating	Reported	Overeating in 20 participants during SGAs treatment.
Garriga et al. (2019)	34	Schizophrenia, schizoaffective, or BD	36.8 ± 12.2	Cohort	25–50 mg/d clozapine, normal weight/overweight	18 weeks	Food craving	FCI-SP and FFQ	-No change in food craving for carbohydrates, proteins, simple sugar, trans fat, and fast-food fats. -The adjusted analyses showed that males and BMI are correlated with fast-food fats craving.
Teff et al. (2015)	30 (10 olanzapine, 10 aripiprazole & 10 placebo)	Healthy	Olanzapine 26.1 ± 3.5, aripiprazole 25.9 ± 4.3, placebo 29.9 ± 7.5	Cohort	Olanzapine & aripiprazole/placebo	12 days	Caloric intake, hunger and fullness	24-h dietary recall, monitoring and calculating food intake & VAS	Caloric intake with olanzapine -Calories consumed after olanzapine exposure comparing to placebo (d: 3.49) Caloric intake with aripiprazole -Calories consumed after aripiprazole exposure comparing to placebo (d: 4.13). Hunger and fullness -No change
Piparva et al. (2011)	74	Psychotic disorders	Not reported	Cohort	Risperidone, olanzapine, aripiprazole, clozapine & quetiapine	<18 months	Appetite	Reported and using NADRPS	-Appetite increase (# 10, 10.75%). Probable offending drugs: olanzapine (# 6), risperidone (# 2) and clozapine (# 2). -Appetite decrease (# 9, 9.67%). Probable offending drugs: risperidone (# 7) and quetiapine (# 2).
Murashita et al. (2005)	7	Schizophrenia	46.3 ± 15.7	Cohort	10.7 mg/d olanzapine	6 months	Appetite	Reported	Appetite increase in (# 6, 85.7%) after 6 months of treatment.
Treuer et al. (2009)	631	Schizophrenia & BD	32.6 ± 12.2	Cohort	11.5 mg/d olanzapine	6 months	Appetite, fullness and dietary habits	Outcomes were reported & the frequency of consumption of certain food groups were monitored by researchers	At 6 months: -49% reported appetite increase. -35% requires a larger amount of food to reach fullness. -26% of who have increased eating frequency tend to consume sweets and candies.
Stip et al. (2012)	24	Schizophrenic	30.04 ± 9.41	Cohort	16 mg/d olanzapine	16 weeks	Eating behaviour	TFEQ	No change in the TFEQ scores after treatment.
Bachmann et al. (2012)	74	Schizophrenia spectrum disorder	19.9 ± 2.3	Cross-sectional	269.8 mg/d clozapine & 15.8 mg/d olanzapine	2.5 ± 1.6 years	Eating behaviour	TFEQ	-Restrained eating behaviours between male and female (d: 0.02). -Disinhibited eating behaviours between male and female (d: 0.02). -Hunger between male and female (d: 0.32). -Total score of the TFEQ between male and female (d: 0.15).
Blouin et al. (2008)	38 (18 SGA, 20 reference)	First psychoses, non-schizophrenic patients	30.5 ± 7.9 SGA and 29.5 ± 6.7 reference	Cross-sectional	Clozapine, olanzapine, risperidone, quetiapine & ziprasidone/healthy controls	>3 months	Eating behaviour, food preference and caloric intake	TFEQ, experimental meals sessions, monitoring and calculating food intake & food preference	-Dietary disinhibition, restraint and hunger behaviours were higher in the treatment group compared to controls. d cohen for: 1) Dietary restraint: 0.68, dieting: 0.67, self-regulation attitudes: 0.11 and avoidance of calorie dense food: 0.91. 2) Dietary disinhibition: 0.78, habitual eating behaviours: 1.02, emotional eating behaviours: 0.60 and the tendency for situational behaviours: 0.0. 3) Hunger: 1.41, internal hunger cues: 0.98 and external hunger cues: 1.25. -No significant difference in caloric intake between groups during experimental meals (dcohen: 0.44). -No significant difference in food preference between groups during experimental meals. d cohen for: 1) carbohydrates %: 0.3. 2) Fat %: 0.02. 3) Protein %: 0.5. 4) Carbohydrates in grams: 0.43. 5) Fat in grams: 0.4. 6) Protein in grams: 0.15 -Hunger was higher than control in the breakfast session.
Boon-Yasidhi et al. (2014)	45	Autism spectrum disorders	8.15 ± 2.98	Cross-sectional	0.94 mg/d risperidone	36.8 ± 27.8 Months	Appetite	ADRS questionnaire developed by investigators	Appetite increase (# 26, 57.8%).
Calarge et al. (2012)	110	Psychiatric diagnosis	11.8 ± 2.9	Cross-sectional	1.4 mg/d risperidone	2.5 ± 1.7 years	Caloric intake and dietary composition	Block FFQ	No correlation between caloric intake and dietary composition with weight.
Calarge and Ziegler (2013)	13 (15 iron deficient, 51 iron depleted, 47 iron repleted)	Non-specified psychiatric disorders	11.6 ± 2.8	Cross-sectional	0.03 mg/kg/d risperidone	>6 months	Caloric intake and dietary composition	Block FFQ	Risperidone treated individuals: -Higher consumption of calories in the iron depleted compared to the iron repleted group (d: 3.16). -Higher consumption of carbohydrates in the iron depleted compared to the iron repleted group (d: 3.6).
de Beaurepaire (2021)	156 (15 Clozapine, 33 olanzapine, 35 risperidone, 24 aripiprazole & 7 amisulpride)	Schizophrenia	41.7	Cross-sectional	Clozapine, olanzapine, aripiprazole, risperidone & amisulpride	<8 weeks	BN, binge eating, BED, and night eating	BN, binge eating or BED according to the diagnostic criteria of DSM-IV, and night eating: “Do you get up at night to eat?”	**Prevalence of EDs in antipsychotics treated individuals:** -No cases of BN. -4% cases of BED. -19% cases of binge eating and 30% of night eating not fulfilling the diagnostic criteria. -BED and night eating were significantly more prevalent in the clozapine and olanzapine group for females (BED, d: 0.85; night eating, d: 1) and not for males. -Lower prevalence of BED in patients treated with olanzapine and clozapine for >2 years when compared with patients treated for < 2years (d:0.85).
Gebhardt et al. (2007)	64 (33 clozapine, 31 olanzapine)	Patients with treatment of psychotic symptoms on clozapine or olanzapine	30.7 ± 13.9	Cross-sectional	Clozapine & olanzapine	>4 weeks	EDs and appetite	QEWP, M-CIDI, NADRPS and an interview.	-Patients fulfilled criteria for EDs after being introduced to clozapine and olanzapine (# 5, 83%, 1 definite and 4 probable). -Deterioration of EDs symptoms after exposure to clozapine and olanzapine. -Higher appetite increase in the clozapine when compared to olanzapine group. -Appetite was reported as extreme (12.5%), very much (20.3%), slightly (35.9%) and not increased (21.9%) by the shole study sample.
Goluza et al. (2017)	93	Schizophrenia	>18	Cross-sectional	Quetiapine, olanzapine & clozapine	NR	Food addiction	YFAS	-25 individuals (26.9%) met the diagnostic YFAS criteria for food addiction. -77% of participants who did not meet food addiction criteria endorsed 3 or more food addiction symptoms such as: 1) repeated unsuccessful efforts to decrease consumption (98%), 2) overeating despite awareness of related health consequences (64%) and 3) persistant behaviour despite the awareness of consequences (58%).
Henderson et al. (2006)	88 (88 SGAs, 723 controls)	Schizophrenia	45 ± 10	Cross-sectional	Olanzapine, clozapine, risperidone, quetiapine & ziprasidone/healthy controls	NR	Caloric intake & dietary composition	Four-day dietary intake & FFQ	Caloric intake -Less calories consumed than general population (d: 0.512). -Higher intake of calories from sweets. Dietary composition -Less carbohydrates (d: 0.502), protein (d: 0.417) and fat (d: 0.371) intakes than general population. -Less fruits, vegetables, grains, meats and dairy products than the US dietary recommendations. -Less than one serving of fruits and vegetables/d -A mean of 4 servings of fat/d.
Jakobsen et al. (2018b)	428	Schizophrenia, schizoaffective psychosis and persistent delusional disorder	38.66 ± 12.4	Cross-sectional	Clozapine, olanzapine & quetiapine	NR	Dietary composition, dietary quality, dietary habits and caloric intake	FFQ, 24 h recall, DQS and Dankost Pro Software	**Caloric intake** -Caloric intake between the study sample and the general population (d: 0.51). -Caloric intake did not differ with exposure to SGA and was not dependant on the type of SGA used. **Dietary habits** -217 (62.4%) participants reported having homecooked or partly homecooked meals during the last 24 h. -The participants' diet consisted of averagely 11.8% added sugars comparing to the recommended consumption of 10%. **Dietary composition** -The average diet of the study population is in line with the dietary recommendations. d cohen is: 0.59 for energy percent from fat, 0 for energy percent from protein and 0.25 for energy percent from carbohydrates. -The study participants consume 120 g of fruit and vegetables daily comparing to a minimum 600 g daily recommended. **Dietary quality** -Scores were not correlated with whether participants treated with SGAs.
Khazaal et al. (2009)	37 (22 SGAs, 15 controls)	Schizophrenia & schizoaffective disorders	30 ± 8.3	Cross-sectional	Olanzapine, clozapine, risperidone & quetiapine/controls	NR	Eating behaviour, hunger & fullness	TFEQ and VAS	**Eating behaviour** -Between group differences in TFEQ scores at baseline (d: 1). **Hunger and fullness** -Significant decrease in hunger after consumption of sweet stimuli (aspartame and sucrose). -Decrease in hunger (d: 0.96) and fullness (d: 1.12) over time.
Kurpad et al. (2010)	73 (9 BE, 64 without BE)	Psychosis	17–65	Cross-sectional	Olanzapine & risperidone	< or >2 years	Binge eating	Reported	Binge eating was seen in 78% of participants treated with risperidone and 22% of participants treated with olanzapine.
Lappin et al. (2018)	24	Schizophrenia & schizoaffective disorder	43.7 ± 11.2	Cross-sectional	Median dose 275 mg/d clozapine & aripiprazole	10.4 ± 4.9 years	Dietary habits	Unpublished, semi-structured food intake questionnaire	-More than half of participants did not consume fruits, vegetables, wholegrains, sugar free dairy products or foods with healthy fats, daily. -Participants reporting choosing fruit (17%), vegetables (17%), wholegrain foods (16%), sugar free dairy products (8%), foods with healthy fats (18%), multiple times a day. -High consumption of unhealthy food choices: empty caloric savoury (55%) and sweet food items (54%), and sweet drinks (37%), once or more daily.
Lundgren et al. (2006)	399	Non-specified psychiatric disorders	40.8 ± 12.7	Cross-sectional	Aripiprazole, olanzapine, quetiapine, risperidone & ziprasidone	NR	NES	NEQ & night eating syndrome History and Inventory (unpublished semi-structured interview)	Participants with NES are using SGAs more frequently than those without the syndrome (d: 0.25).
Morell et al. (2019)	301	Non-specified psychiatric disorders	44.4 ± 12.3	Cross-sectional	Median DDD of 1 mg/d SGAs	Median 3.0, range 0–36 years	dietary habits	Eating behaviour was assessed using a targeted 10-question, picture-guided, food intake questionnaire	High consumption of soft drinks and empty caloric food items.
Ngai et al. (2018)	54 (20 risperidone, 16 quetiapine, 18 controls)	Non-specified psychiatric disorders	9–18	Cross-sectional	Risperidone & quetiapine/controls	>3 months	Appetite	VAS	-Increased drive to eat during OGTT in the risperidone group compared to controls for 2 of the 4 questions asked: “How strong is your desire to eat?” (d: 1.03) and “How much food do you think you can eat?” (d: 0.74). -No difference between the quetiapine treated participants and controls.
Nuntamool et al. (2017)	82	Autism disorders	Median age: 11 years (IQR: 9.00–14.00)	Cross-sectional	Median dose 0.75 risperidone	median 67.90 (52.53–90.93) months	Appetite	Reported	Appetite increase in10 participants (16.39%) of the stable group arm.
Peled et al. (2020)	43	Cancer	12.1 ± 5.2	Cross-sectional	3.5 mg/d olanzapine & 0.8 mg/d risperidone	NR	Appetite	Retrospectively from patients' records	Appetite decrease -63% of patients with depressed mood. -27% of patients without depressed mood. -15 (60%) of participants on olanzapine. -8 (44%) of participants on risperidone.
Platzer et al. (2020)	232 (139 BD, 93 HC)	BD	Male 46.5 ± 14.1, female 43.9 ± 13.7	Cross-sectional	Olanzapine & quetiapine	NR	Food craving	FCI	Higher sweets craving in the olanzapine and quetiapine groups than other types of medication (d: 0.413)
Pozzi et al. (2013)	56	Non-specified psychiatric disorders	<18	Cross-sectional	Risperidone, aripiprazole, olanzapine & quetiapine	1 year	Appetite	Retrospective analysis of ADRS reported and Naranjo	-Antipsychotics linked to appetite increase and weight gain in 43% of participants. -Appetite increase with risperidone (5 of 11 participants) and aripiprazole (1 of 3 participants).
Qurashi et al. (2015)	56	Non-specified psychiatric disorders	37.9 ± 10.6	Cross-sectional	349 mg/d clozapine	>3 months	Appetite	Five-point scale	Change in appetite with clozapine compared to previous antipsychotics (Much worse: 0; worse: 11; no different: 22; better: 17; and much better: 6).
Sentissi et al. (2009)	153 (93 SGA, 27 FGA, 33 controls)	Schizophrenic	33.1 ± 8.7	Cross-sectional	Clozapine, olanzapine, amisulpride, risperidone & aripiprazole	>3 months	Eating behaviour	TFEQ and DEBQ	**DEBQ (SGAs and controls) d cohen:** -External eating: 0. -Emotional eating: 0.16. -Restriction: 0. **DEBQ (SGAs and FGAs) d cohen:** -External eating: 0.52, p = 0.035. -Emotional eating: 0.33. -Restriction: 0.26. **TFEQ (SGAs and controls) d cohen:** -Restriction: 0.04. -Disinhibition: 0.05. -Hunger: 0.09. **TFEQ (SGAs and FGAs) d cohen:** -Restriction: 0.23. -Disinhibition: 0.47. Hunger: 0.29.
Teasdale et al. (2018)	93	Psychosis	21.4 ± 2.9	Cross-sectional	Chlorpromazine equivalent 242 mg/d risperidone, aripiprazole, olanzapine, quetiapine, paliperidone, clozapine, amisulpride & ziprasidone	A median for 8 months, IQR 11 months	Caloric intake and dietary habits	Caloric intake based on AGHE, Schofield equation and the estimation of food servings	**Caloric intake** -Caloric intake higher than energy requirements (d: 3.6085). -Olanzapine treated patients reported caloric intake compared to amisulpride (d: 1.53), risperidone (d: 0.94) and aripiprazole (d: 1). -No correlation between drug dosage (d: 0.01) and duration of treatment (d = 0.04) with caloric intake. **Dietary habits** -Highly consumed food items includes grains (33%), empty caloric (31%), high protein containing (17%), added fat (6%), dairy (6%), fruits (4%), and vegetables (3%). -Higher intakes of empty caloric food items comparing to the Australian dietary recommendation (d: 0.71). -Lower intakes of vegetables (d: 0.71), dairy products (d: 0.71) and unsaturated fats (d: 0.71) comparing to the Australian dietary recommendations. -Grains: (19% wholegrain and 81% refined products).
Theisen et al. (2003)	74 (17 Olanzapine, 57 Clozapine)	Non-specified psychiatric disorders	19.8 ± 2.2	Cross-sectional	Clozapine & olanzapine	2.4 ± 1.6	BE, BED & BN	Screening question for binge eating, the modified version of QEWP and the self-report instrument based on DSM-IV	BE in the clozapine group: -Negative (# 31, 54.4%). -Positive (# 26, 45.5%). BE in the olanzapine group: -Negative (# 6, 35.3%). -Positive (# 11, 64.7%). Participants meeting diagnostic criteria for BED: -Clozapine group: # 5, 8.8%. -Olanzapine group: # 4, 23.5%. Participants meeting diagnostic criteria for BN: -Clozapine group: # 2, 3.5%. -Olanzapine group: # 1, 5.9%.
Yektaş and Tufan (2018)	212	Non-specified psychiatric disorders	13.0 ± 4.1	Cross-sectional	Median 1 mg/d risperidone, 5 mg/d aripiprazole, 100 mg/d quetiapine & male 15 female 10 mg/d olanzapine & male 2.5 female 3.5 haloperidol	1 year	Appetite	Reported	Appetite increase is of the most commonly seen drug adverse effects.
Dell'Osso et al. (2012)	30	Mood disorders	50.1 ± 14.39	Prospective	365 mg/d quetiapine IR, 373 mg/d quetiapine XR	6 weeks	Appetite	Reported	Appetite increase with weight gain in 8.4% of participants.
Masi et al. (2013)	40 (22 responders, 18 non-responders)	BD	14.9 ± 2.0	Prospective	258 mg/d quetiapine	3 months	Appetite	Reported	Appetite increase (# 8, 20%).
Sharma et al. (2014)	100	Schizophrenia, schizoaffective disorder, schizophreniform disorder, brief psychotic disorder, mania first episode & unspecified psychosis	Male 28.1 ± 7.07, female 31.3 ± 8.1	Prospective	Risperidone, olanzapine, aripiprazole, amisulpride & clozapine	12 weeks	Appetite	VAS	Appetite increase in all follow ups points.
Basgul (2014)	41	Non-specified psychiatric disorders	14.9 ± 2.6	Retrospective	10 mg/d aripiprazole	1 month	Appetite	Reported	Appetite increase (# 2).
Coskun et al. (2011)	25	Disruptive Behaviour disorders	45.79 ± 11	Retrospective	Maximum mean dose 0.52 risperidone	18.87 ± 15.19 weeks	Appetite	Reported	Appetite increase: -Mild: 5. -Moderately severe: 5. -Overall: 10, 40%. Appetite decrease: -1, 4%.
Demirkaya et al. (2017)	14	Conduct disorder	13.9 ± 2.9	Retrospective	Initial dose 25 mg/d risperidone	Mean 3.1 months 1.5–8 months	Appetite	Reported	Appetite increase (# 1) with no weight gain.
Moore et al. (2013)	115	Eating disorder	13 ± 1.60	Retrospective	56 mg/d quetiapine, 6.8 mg/d olanzapine & 1 mg/d risperidone	2 ± 1.52 years	Binge eating	Routine assessment using a standardised assessment document	-SGAs associated with new onset of binge eating. -Patients with worsening binge eating (# 2). -Patients with concern that olanzapine may have worsened their binge eating (# 4). -Participants on SGAs have higher frequency of binge eating when compared to other medications. -Odds ratio of new binge eating onset is 3.5, with SGAs use.
Okamoto et al. (2019)	80	Cancer	60.4 ± 15.7	Retrospective	2.28 mg/d olanzapine	3 days	Caloric intake	Monitoring and calculating food intake	-Caloric intake 149% higher than baseline. -Caloric intake 143% higher than baseline in the 40 nausea-free patients. -Caloric intake 124% higher in the 3rd day after exposure to 1.5 mg/d olanzapine in the 13 nausea-free patients. -No difference in caloric intake between a dose of 1.5 mg/d or more.

Shaded rows indicate the inclusion of children and adolescents. ^a^ age in mean ± standard deviations were substituted with age range or median and interquartile range if they were not reported. ^b^ Mean dose was substituted with range, maximum, initial, median or fixed doses when not reported. ^c^ Duration of intervention in interventional studies was substituted with length of follow up in observational studies reported in mean ± standard deviations or range. #: Number of participants experiencing outcomes; AAP: Atypical Antipsychotic; AD: Autistic Disorder; AD: Antidepressants; ADHD: Attention deficit hyperactivity disorder; ADRS: Adverse Drug Reactions; AGHE: Australian guide to healthy eating; AN: anorexia nervosa; APD: Antipsychotic drugs; ASD: Autism Spectrum Disorder; BD: Bipolar Disorder; BE: Binge Eating; BED: Binge Eating Disorder; Block FFQ: Block Kids Food Frequency Questionnaire; BMI: Body Mass Index; BN: Bulimia Nervosa; CGI-S: Global Impression-Severity; CI: Confidence interval; d: Day; df: difference; DOTES: Dosage Record Treatment Emergent Symptom Scale; DQS: Dietary Quality Score; DRI: Dietary Reference Intake; DSM-IV: Diagnostic and Statistical Manual of Mental Disorders-Fourth edition; EBA: Eating Behaviour Assessment; FCI: Food craving inventory; FCI-SP: Food Craving Inventory-Spanish Version; FFQ: Food Frequency Questionnaire; FGA: first generation antipsychotics; g: gram; HC: healthy controls; HR: hazard ratio; IR: immediate release; Kcal: kilocalories; KJ: Kilojoules; LBM: Lean Body Mass; M-CIDI: M-Composite International Diagnostic Interview; mcg: microgram; mg: milligram; MS: Mood Stabilizers; MUFA: Monounsaturated Fatty Acids; NADRPS: Naranjo Adverse Drug Reaction Probability Scale; NEQ: Night Eating Questionnaire; NPI: Neuropsychiatric inventory; NR: Not reported; NRS: Numerical Rating Scale; NS: not significant; ODO: Orally Disintegrating Olanzapine; OGTT: oral glucose tolerance test; OR: Odds Ratio; PUFA: Polyunsaturated Fatty Acids; QEWP: Questionnaire on Eating and Weight Patterns; r: correlation coefficient; RCT: Randomized Controlled Trial; RDA: Recommended Dietary Allowance; SGA: second generation antipsychotics; SOT: Standard Olanzapine Tablets; TD: Tourette's disorder; TD: Tic Disorder; TFEQ: Three-factor eating questionnaire; VAS: Visual Analog Scale; XR: extended-release; YFAS: Yale Food Addiction Scale.

a age in mean ± standard deviations were substituted with age range or median and interquartile range if they were not reported.

b Mean dose was substituted with range, maximum, initial, median or fixed doses when not reported.

c Duration of intervention in interventional studies was substituted with length of follow up in observational studies reported in mean ± standard deviations or range.

#### 3.1.1 Participants

The sample included the following populations: in- and out-patients diagnosed with schizophrenia (n = 27); autistic disorders (n = 15); unspecified psychiatric diagnoses (n = 14); schizoaffective disorder (n = 13); bipolar disorders (n = 11); schizophreniform disorder (n = 7); psychotic disorders (n = 6); pervasive developmental disorders (n = 3); conduct and disruptive behavior disorders (n = 3); tic disorder (n = 3); alcohol dependence disorder (n = 2); transient psychotic disorders (n = 1); mental retardation (n = 1); Alzheimer's disease (n = 1); Tourette's syndrome (n = 1); mood disorders (n = 1); depression (n = 1); eating disorders (n = 1); transient psychotic disorder (n = 1); attention deficit hyperactivity disorder (n = 1); borderline personality disorder (n = 1); first episode mania (n = 1); and cancer (n = 3). Other studies also included healthy individuals treated with SGAs (n = 6).

Most of the studies reported outcomes of adults (n = 56) either alone or in combination with sample of children, adolescents, adults or senior adults. Others included only children (n = 35), adolescents (n = 37) or senior adults (n = 18). The mean age was 26 ± 15 years and ranged from 2 to 85 years, reported by 83 studies. For more details about the characteristics of participants of included studies see Table 1.

#### 3.1.2 Intervention

Olanzapine was the most widely used SGA in 33 studies followed by risperidone in 28 studies. The less frequently used SGAs were aripiprazole (n = 11), clozapine (n = 7), quetiapine (n = 7), ziprasidone (n = 1), asenapine (n = 1), ileoperidone (n = 1) and/or other mixes of SGAs. Three studies included patients treated with SGAs in combination with other type of drugs such as mood stabilizers, benzodiazepine, antidepressants and FGAs (de Beaurepaire, 2021; Lundgren et al., 2006; Morell et al., 2019; Teasdale et al., 2018). Only data related to SGAs were outlined in this review. All drug related data including drug dosages can be found in more detail in Table 1.

#### 3.1.3 Outcomes and measures

Most of the studies reported eating- and appetite-related outcomes using subjective assessment tools. These include data reported from patients, carers or measured by health care practitioners, visual analogue scales, dosage records and treatment emergent symptom scales and adverse drug reactions checklists (n = 55). Diagnostic tools for studies reporting eating disorder symptomatology and diagnoses (n = 7) included the M-composite international diagnostic interview, criteria for the Diagnostic and Statistical Manual of mental disorders-Fourth edition (DSM-IV), the night eating questionnaire and other screening questions and self-report instruments based on DSM-IV criteria (de Beaurepaire, 2021; Gebhardt et al., 2007; Kluge et al., 2007; Kurpad et al., 2010; Lundgren et al., 2006; Moore et al., 2013; Theisen et al., 2003).

Other studies used validated questionnaires to measure outcomes of interest such as the Food Craving Inventory to measure food craving (n = 4) (Abbas and Liddle, 2013; Bobo et al., 2011; Garriga et al., 2019; Platzer et al., 2020), the Yale Food Addiction Scale to measure food addiction (n = 1) (Goluza et al., 2017) and the Dietary Quality Score to measure dietary quality (n = 2) (Jakobsen et al., 2018a, 2018b).

Eating behaviour was measured using the three-factor eating questionnaire (TFEQ) and the Dutch Eating Behaviour Questionnaire in 7 studies (Bachmann et al., 2012; Blouin et al., 2008; Bobo et al., 2011; Khazaal et al., 2009; Mathews et al., 2012; Sentissi et al., 2009; Stip et al., 2012). Caloric intake and dietary composition was measured by either monitoring and calculating food intake manually by investigators (n = 7) (Ballon et al., 2018; Blouin et al., 2008; Fountaine et al., 2010; Gothelf et al., 2002; Okamoto et al., 2019; Roerig et al., 2005; Teff et al., 2013) or by using standardized tools such as the Food Frequency Questionnaire, 24-h dietary recall or built-in software such as the Dankost Pro (n = 7) (Calarge et al., 2012; Calarge and Ziegler, 2013; Daurignac et al., 2015; Henderson et al., 2006; Jakobsen et al., 2018b; Kluge et al., 2007; Lindsay et al., 2006; Teff et al., 2015). For more details about outcomes and outcome measures used in eligible studies, refer to Table 1.

### 3.2 Characteristics of studies included in the meta-analyses

A total of three studies were eligible for inclusion in the meta-analysis to compare the three eating behaviour subscales of the TFEQ cross-sectionally after treatment (Blouin et al., 2008; Khazaal et al., 2009; Sentissi et al., 2009). These studies included adult participants (18–65 years old) diagnosed with first episode psychoses (non-schizophrenia), schizophrenia and schizoaffective disorders. Independent analyses were conducted for each of the eating behaviour subscales: disinhibition, restraint and hunger.

Four additional studies also used the TFEQ but were ineligible for inclusion in the meta-analyses and thus were described narratively (Bachmann et al., 2012; Bobo et al., 2011; Mathews et al., 2012; Stip et al., 2012). See supplementary material S.6 for a qualitative appraisal of these studies.

Results from the eight studies that were combined using odds ratios were all longitudinal, reporting binary outcomes pertaining to appetite increase (Aman et al., 2005; Black et al., 2014; Guardia et al., 2004; Litten et al., 2012; McCracken et al., 2002; Roerig et al., 2005; Snyder et al., 2002; Srivastava et al., 2012). Diagnoses of participants included in this analysis were autism disorder (n = 2), alcohol dependence disorder (n = 2), conduct and disruptive behaviour disorder (n = 1), bipolar disorder (n = 1), borderline personality disorder (n = 1) and one study included healthy participants. Three studies included children aged from 5 to 12 years old (Aman et al., 2005; McCracken et al., 2002; Snyder et al., 2002), and the remaining included adults with an age range from 18 to 60 years old (Black et al., 2014; Guardia et al., 2004; Litten et al., 2012; Roerig et al., 2005; Srivastava et al., 2012). For more details about the characteristics of studies and participants included in the meta-analyses and odds ratio see Table 2.

**Table 2:** Summary of studies included in the meta-analyses and odds ratio.

Publication	Sample size	Sample characteristics	Age (years)^a^	Agent/comparison^b^	Duration
**Meta analyses**					
Blouin et al. (2008)	38 (18 SGA, 20 controls)	First psychoses, non-schizophrenic patients	30.5 ± 7.9 SGA	Clozapine, olanzapine, risperidone, quetiapine & ziprasidone/controls	>3 months
			29.5 ± 6.7 reference		
Khazaal et al. (2009)	37 (22 SGAs, 15 controls)	Schizophrenia & schizoaffective disorders	30 ± 8.3	Olanzapine, clozapine, risperidone & quetiapine/controls	NR
Sentissi et al. (2009)	153 (93 SGA, 27 FGA, 33 controls)	Schizophrenic	33.1 ± 8.7	Clozapine, olanzapine, amisulpride, risperidone & aripiprazole/controls	>3 months
**Odds ratio**					
McCracken et al. (2002)	101 (49 Risperidone, 51 Placebo)	Autism disorder	8.8 ± 2.7	1.8 mg/d risperidone/placebo	24
Snyder et al. (2002)	110 (53 Risperidone, 57 placebo)	Conduct and disruptive behaviour disorder	5–12	0.98 mg/day risperidone/placebo	7
Guardia et al. (2004)	60 (29 Olanzapine, 31 Placebo)	Alcohol-dependence disorder	18–60	7.54 mg/d olanzapine/placebo	12
Aman et al. (2005)	101 (49 Risperidone, 52 placebo)	Autism disorder	8.8 ± 2.7	<20–44.9 kg: 0.5 mg titrated to 2.5 mg/day	26
				>45 kg: 0.5 mg titrated to 3.5 mg/day risperidone/placebo	
Roerig et al. (2005)	48 (16 olanzapine, 16 Risperidone/16 placebo)	Healthy	18–60	8.75 mg/d olanzapine & 2.875 mg/d risperidone/placebo	2
Litten et al. (2012)	218 (113 placebo 105 Quetiapine)	Alcohol-dependence disorder	18–64	Target dose of 400 mg/d quetiapine/placebo	17
Srivastava et al. (2012)	50 (25 olanzapine & 25 placebo)	Bipolar disorder	40.8 ± 11.5	Olanzapine/placebo^d^	1
Black et al. (2014)	111 (33 low-dose, 33 moderate-dose, 29 placebo)	Borderline personality disorder	18–45	150–300 mg/d quetiapine/placebo	8

Shaded rows indicate the inclusion of children and adolescents. All studies included in the odds ratio calculation are randomized controlled trials. Studies included in the eating behaviour meta-analyses are cross-sectional studies. NR: Not reported.

a Age range in years was reported when mean ± standard deviation was not available.

b Mean dose of the treatment agent was reported. c Represents the duration of intervention in weeks.

d Drug dose was not mentioned.

### 3.3 Quality assessment

Overall, the quality ratings for all the 92 studies were of acceptable quality and thus all were included in our review. Many studies have used validated outcome measures while others used newly developed or non-validated tools to measure outcomes of interest. Other limitations found in the eligible studies included missing raw data and effect size presentations, uncertainties in the diagnosis of the study sample, undetailed inclusion and exclusion criteria, small sample sizes, mixed types of SGA exposure and limited follow up periods due to the nature of some studies such as cross-sectional studies. For more details about the quality assessment of included studies, refer to S.5.

### 3.4 Effects of SGAs on eating-related outcomes

#### 3.4.1 Hunger, appetite, food craving and addiction

##### 3.4.1.1 Cross-sectional meta-analysis of TFEQ hunger subscale

**Fig. 2:**
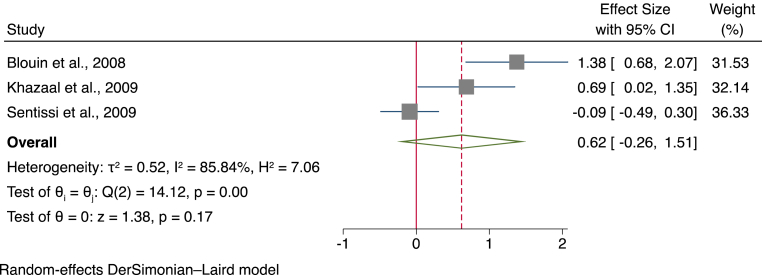
Forest plot for the hunger subscale of the three-factor eating questionnaire.

Data from a total of three studies, using a total sample of 201 participants (133 treated with SGAs and 68 controls) were analysed to compare the hunger subscales of the TFEQ. Overall, results suggested that there was a moderately greater score in the hunger subscale of the TFEQ in participants given SGAs (SMD = 0.62, 95% CI ]-0.26, [1.51, *p* = 0.17) (see Fig. 2 for a forest plot).

##### 3.4.1.2 Odds ratio for a change in appetite following treatment with SGAs

**Fig. 3:**
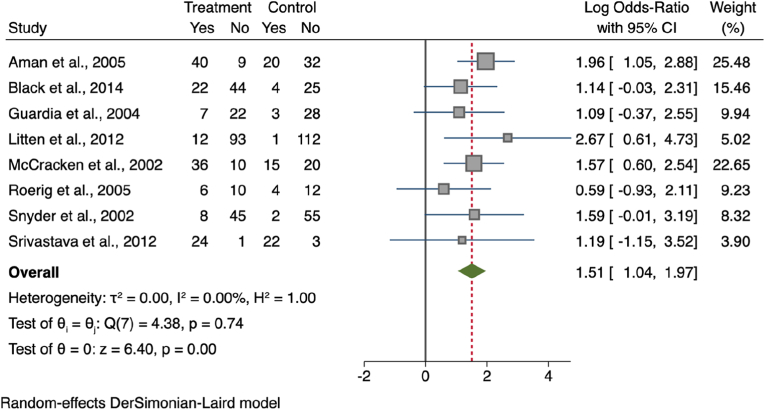
Odds ratio for appetite increase.

Fig. 3 shows the calculated odds ratio of appetite increase versus no change in appetite between 392 participants on SGAs and 374 controls from eight studies (Aman et al., 2005; Black et al., 2014; Guardia et al., 2004; Litten et al., 2012; McCracken et al., 2002; Roerig et al., 2005; Snyder et al., 2002; Srivastava et al., 2012). Overall, the odds ratio for an increase in appetite after SGA treatment was 1.51 and the difference between the treatment and control groups was statistically significant (95% CI [1.04, 1.97]; z = 6.40; *p* < 0.001).

**Fig. 4:**
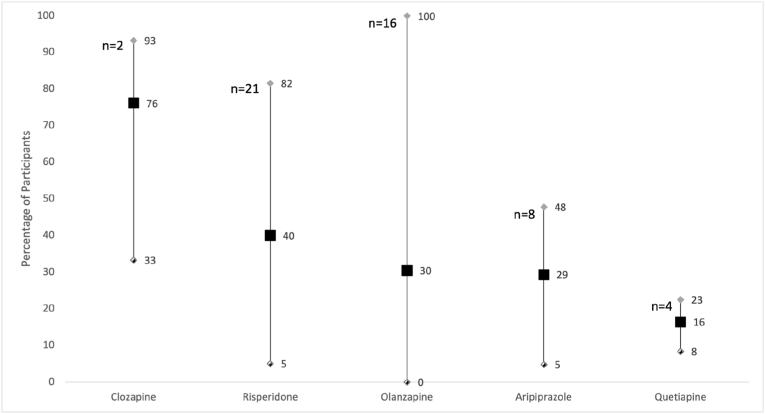
Weighted mean percentage of participants reporting an increase in appetite. Self-reported appetite increase seen in participants treated with different SGA types. The chart indicates the weighted mean percentages with the minimum and maximum percentages obtained from available data. n = number of studies included in the weighted mean percentages calculations.

Additionally, Fig. 4 depicts the weighted mean percentages of participants reporting appetite increase with SGA use, across all studies included in the systematic review and meta-analysis, per type of SGA. 51 studies were included, of which the numbers of studies examining each SGA specific drug were: 2 for clozapine; 21 for risperidone; 16 for olanzapine; 8 for aripiprazole and 4 for quetiapine. All included studies reported appetite increase experienced with these SGAs.

##### 3.4.1.3 Food craving

The effect sizes between the SGA and control groups in food cravings, which were calculated from the data of (Abbas and Liddle, 2013), were slightly higher for the fat (*d* = 0.24) and carbohydrate (*d* = 0.18) craving. Minimal differences were seen for the general foods (*d* = 0.11), sweets (*d* = −0.05), and fast-food fat (*d* = −0.16) cravings (Abbas and Liddle, 2013).

##### 3.4.1.4 Food addiction

Only one study reported food addiction as an outcome in patients treated with SGAs (Goluza et al., 2017), finding that approximately 27% of participants treated with quetiapine, olanzapine and clozapine met the diagnostic criteria for food addiction and 77% had at least 3 food addiction symptoms.

For more details about the qualitative appraisal of studies investigating hunger, appetite, food craving and addiction, see supplementary material S.6.

#### 3.4.2 Dietary disinhibition, binge eating and loss of control over eating

##### 3.4.2.1 Cross-sectional meta-analysis of TFEQ dietary disinhibition subscale

**Fig. 5:**
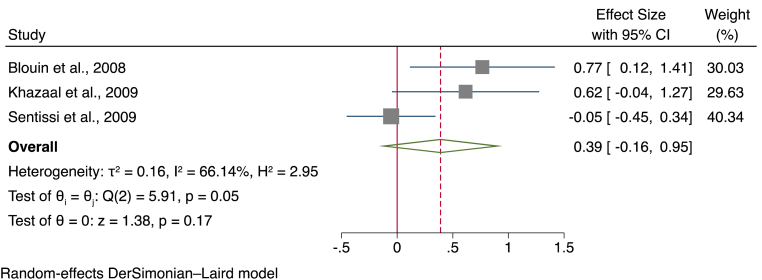
Forest plot for the dietary disinhibition subscale of the three-factor eating questionnaire.

Meta-analysis was performed using data from a total of three studies and a total sample of 201 participants (133 treated with SGAs; 68 controls) to compare the disinhibited eating subscales of the TFEQ. Results of this analysis revealed a small increase in disinhibited eating in patients treated with SGAs (SMD = 0.40, 95% CI ]-0.17, 0.97 [, *p* = 0.169) compared to controls and an overall substantial heterogeneity (I^2^ = 66.43%) (See Fig. 5 for a forest plot). For more details about the qualitative appraisal of studies investigating disinhibited eating, binge and loss of control over eating, see supplementary material S.6.

#### 3.4.3 Dietary restraint

##### 3.4.3.1 Meta-analysis of TFEQ dietary restraint subscale

**Fig. 6:**
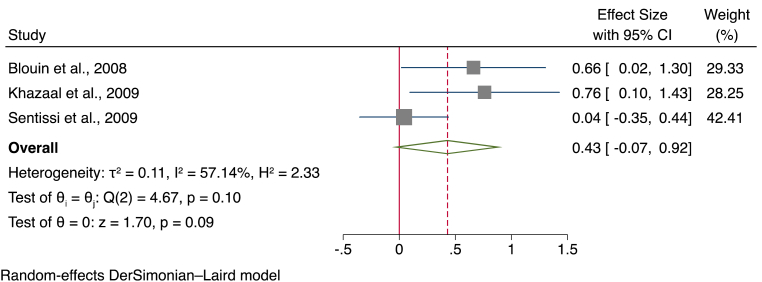
Forest plot for the restraint subscale of the three-factor eating questionnaire.

Data from a total of three studies, using a total sample of 201 participants of which 133 treated with SGAs and 68 controls were analysed to compare the restrained eating subscales of the TFEQ. Overall, this meta-analysis showed a small increase in restrained eating in patients treated with SGAs (SMD = 0.43, 95% CI ]-0.07, [0.94, *p* = 0.091) compared to controls with substantial heterogeneity between studies (I^2^ = 57.68%) (see Fig. 6 for a forest plot).

#### 3.4.4 Satiety and fullness

A total of 5 studies measured satiety and fullness (Ballon et al., 2018; Khazaal et al., 2009; Roerig et al., 2005; Teff et al., 2015; Treuer et al., 2009) in participants treated with SGAs.

One study found an increase in fullness (Khazaal et al., 2009) while two others reported no effect of olanzapine, aripiprazole and risperidone on satiety and fullness in an experimental food laboratory environment (Roerig et al., 2005; Teff et al., 2015). Treuer et al. (2009), however, found that approximately 35% of participants treated with olanzapine needed a larger amount of food in order to feel full.

#### 3.4.5 Caloric intake

A total of 17 studies reported caloric and food intake in patients treated with SGAs (Ballon et al., 2018; Blouin et al., 2008; Calarge et al., 2012; Calarge and Ziegler, 2013; Cicala et al., 2020; Daurignac et al., 2015; Fountaine et al., 2010; Gothelf et al., 2002; Henderson et al., 2006; Jakobsen et al., 2018b; Kluge et al., 2007; Lindsay et al., 2006; Okamoto et al., 2019; Roerig et al., 2005; Teasdale et al., 2018; Teff et al., 2013, 2015).

Caloric intake of participants treated with SGAs was increased in many eligible studies in our review (Cicala et al., 2020; Fountaine et al., 2010; Gothelf et al., 2002; Kluge et al., 2007; Okamoto et al., 2019; Roerig et al., 2005). However, others reported reductions in calories consumed with SGA treatment (Henderson et al., 2006; Jakobsen et al., 2018b). Caloric intake was higher than requirements in Lindsay et al. (2006) and Teasdale et al. (2018)'s sample. Interestingly, Teasdale et al. (2018) found that treatment with olanzapine relative to other SGA monotherapy resulted in an increase in caloric intake. See Table 1 for detailed summary about the results.

An increase in food intake after the introduction of olanzapine was noted in only one laboratory-based experimental meal study (Ballon et al., 2018).

Calarge and Ziegler (2013) found a relationship between iron status and caloric intake in that the iron depleted group ate more with risperidone.

#### 3.4.6 Dietary composition, quality and eating habits

Dietary composition and quality of participants treated with SGAs were examined in eight eligible publications (Ballon et al., 2018; Calarge et al., 2012; Calarge and Ziegler, 2013; Daurignac et al., 2015; Gothelf et al., 2002; Henderson et al., 2006; Jakobsen et al., 2018b; Lindsay et al., 2006) with varied results.

Assessing macronutrients consumption revealed that the intake of carbohydrates in three studies (Ballon et al., 2018; Calarge and Ziegler, 2013; Lindsay et al., 2006), protein in two (Ballon et al., 2018; Lindsay et al., 2006) and fat in three studies (Ballon et al., 2018; Henderson et al., 2006; Lindsay et al., 2006) was increased with SGAs. Conversely, the consumption of macronutrients did not change from baseline (Gothelf et al., 2002), was in line with the dietary composition of the general populations (Jakobsen et al., 2018b) or lower than controls (Henderson et al., 2006).

Poor diet quality was detected in schizophrenic patients when compared to controls, and this did not change after treatment with SGAs in two studies (Jakobsen et al., 2018a, 2018b). For detailed qualitative appraisal of outcomes relating to dietary composition, quality and eating habits see S.6.

## 4 Discussion

The present study systematically assessed and meta-analysed outcomes relating to eating cognitions, behaviours and emotions in individuals treated with SGAs regardless of their underlying psychiatric diagnosis. A total of 92 studies were included in this systematic review encompassing participants from all age groups.

Both quantitative and qualitative synthesis of a broad range of study designs revealed an increase in appetite and hunger in participants treated with SGAs. Individuals exposed to SGAs were approximately 1.51 times more likely to experience an increase in appetite relative to controls. Moreover, a small increase in restrained and disinhibited eating were found in studies using standardised instruments although it is noteworthy that the largest study included found no effect (Sentissi et al., 2009). A small sized increase in restrained dietary behaviours in the SGA-treated participants was manifest, even though deliberate attempts to restrict dietary intake for the purpose of controlling weight are not usually a characteristic of this patient population (Bellisle, 2009).

Outcomes relating to the drive to eat including food craving and addiction were also considered in this review as a component of appetite control. The effect size for craving was slightly higher for fat (d = 0.38) and carbohydrates (d = 0.44) cravings in the SGA treated compared with the control groups (Abbas and Liddle, 2013).

Notably, one study in our review found that 27% of participants treated with SGAs met diagnostic criteria for food addiction and 77% demonstrated at least 3 markers of addiction to processed and energy-dense food (Goluza et al., 2017). However, more research is necessary into the relationship between SGA use and food addiction in order to substantiate these findings.

Studies on eating behaviours, including the quantity and quality of diets consumed by patients revealed interesting yet limited findings in our review. The caloric intake assessment showed that olanzapine was the most common SGA to cause increased food consumption, followed by clozapine, risperidone and aripiprazole (Cicala et al., 2020; Kluge et al., 2007). The dietary patterns including dietary composition (food source) and habits revealed some evidence for an increased preference for sweets, sugary food/drinks, and energy dense savoury foods (Henderson et al., 2006; Jakobsen et al., 2018b; Kluge et al., 2007; Lappin et al., 2018; Morell et al., 2019; Platzer et al., 2021).

### 4.1 Strength and limitations

To the best of our knowledge, our article is the first systematic review and meta-analysis investigating eating cognitions, emotions and behaviours in individuals treated with SGAs. Selection bias was mitigated by involving two independent reviewers during the screening, data extraction and quality assessment procedures.

Given the high heterogeneity in study designs, the limited number of interventional studies investigating the parameters of interest, we were only able to infer cautious conclusions. The high level of heterogeneity in the reporting of outcomes limited the number of studies eligible for inclusion in a meta-analysis and limited further investigations looking at the effect of drug dosage and treatment duration on meta-analytic findings. Crucially, there were insufficient studies included in the meta-analyses to allow us to conduct a meta-regression extension to our results as it has been indicated that ten or more studies are required for a meta-regression to be appropriate (Schmid et al., 1998).

One of the limitations of our review is including only English articles mostly from the United States which might have caused missing some important findings from non-English publications. Limitations also included the heterogeneity of outcome measures used from subjective, objective and validated questionnaires for each parameter of interest.

### 4.2 Clinical implications

The findings from this study suggest that some SGAs may impact on appetite regulation leading to overeating with resultant weight gain and problems associated with obesity, such as cardiovascular diseases and metabolic disturbances (Davis et al., 2020; Khosravi, 2020; Sankaranarayanan et al., 2021). In addition to the social adversity associated with severe mental illness (Compton et al., 2020), which may contribute to poor dietary patterns, it is possible that there is an interaction between the increase in appetite and our current food environment, which includes easily available, highly palatable and ultra-processed foods that may contribute to the development of food addiction (Küçükerdönmez et al., 2019). These disturbed dietary patterns have been shown recently to be greatly correlated with the risk of developing life-threatening illnesses (Wang et al., 2022).

The use of SGAs may also impact on impulsivity which may contribute to the development of maladaptive eating behaviours such as emotional, restrained, or disinhibited eating, as an addition to the weight gain and metabolic disturbance side effects of the drugs (Moe et al., 2016). Disinhibited eating of fat- and carbohydrate-rich foods may produce rapid weight gain in a short duration of time (de Beaurepaire, 2021; Kobayashi and Takano, 2018). This was commonly noted in patients exposed to the SGAs olanzapine and clozapine (Gebhardt et al., 2007). While not all patients on SGAs develop these side effects, those who do may benefit from additional medical support that may include lifestyle modifications and pharmacological interventions. Further large-scale interventional studies investigating the disinhibited eating associated with the use of SGAs, especially the ones linked with high risk of alteration in metabolic function such as olanzapine and clozapine, are needed in order to develop practical advice in modifying the altered dietary patterns for this patient population.

Available options to prevent and overcome SGA-related metabolic side effects may include choosing an antipsychotic with lower risk of metabolic alterations, involving patients in psychological, lifestyle and behavioural interventions, switching to different antipsychotic or adding adjunctive therapies to antipsychotics such as amantadine or topiramate (Hasan et al., 2019; Holt et al., 2019; NICE, 2014). However, some of the above options might not always be feasible as certain drugs are needed to control the intractable symptoms caused by certain psychiatric disorders. For example, some patients on a clozapine regimen cannot be switched back to a lower affinity SGA especially when clozapine was prescribed as the last remaining treatment option after multiple attempts with other SGAs (Correll and Howes, 2021).

There may be a promising role of some pharmacological treatments such as the glucagon-like peptide receptor agonists, liraglutide and semaglutide for the purpose of controlling weight gain (Shi et al., 2022). The latter is believed to be a practice-changing invention for weight control due to its low risk-benefit ratio seen in recent clinical trials (Wilding et al., 2021; Rubino et al., 2021; Davies et al., 2021; Wadden et al., 2021). Evidence also exists regarding the weight control benefits of some antidiabetic drugs such as metformin (Maayan et al., 2010; Seifarth et al., 2013; Chukir et al., 2021). Reboxetine, bupropion and exenatide were specifically tested as an adjunctive treatment for some SGAs and has shown clinical benefits in lessening the severity of weight gain caused by SGAs (Poyurovsky et al., 2003, 2007; Weizman et al., 2021; Siskind et al., 2018). Although the addition of some adjunctive drugs to SGAs were tested, and some have shown promising effects to alleviate antipsychotic-induced metabolic disturbances, further investigations are yet warranted to identify the most suitable options (Larsen et al., 2017; Lee et al., 2021). Assessing and managing changes in eating behaviours, cognitions and emotions may be key to managing increased appetite and food craving in patients treated with SGAs. The management of increased appetite and weight gain may include psychotherapeutic, psychopharmacological and dietary approaches as well as physical exercise (Taylor et al., 2012; Blundell et al., 2015; Mann et al., 2007; Jackson et al., 2015).

It is worthwhile mentioning the possible therapeutic role of SGAs for patients with eating disorders, specifically, anorexia nervosa, where weight gain remains a challenge (Williams et al., 2021). A systematic review and meta-analysis has shown a positive effect on weight gain (Han et al., 2022). However, we found no studies that examined changes in eating behaviours in this patient population for this review. Therefore, further investigations are warranted.

## 5 Conclusion

The results of the current systematic review and meta-analysis find increases in hunger and appetite in patients treated with SGAs with a tendency towards fat and carbohydrate cravings. Also, there is a small increase in dietary disinhibited behaviours in the SGA treated group. These factors may contribute to weight gain during treatment with antipsychotic drugs. Early detection of altered eating behaviours and appetite increase may allow preventative approaches such as a switch in medication and lifestyle interventions to mitigate this problem.

## Role of the funding source

The funders, the Saudi Arabian Government, the Medical Research Council, the MRC, and the NIHR BRC had no influence on the study design, data collection and evaluation of the data.

## Declaration of competing interest

The authors declare that they have no known competing financial interests or personal relationships that could have appeared to influence the work reported in this paper.

## References

[bib1] Abbas M.J., Liddle P.F. (2013). Olanzapine and food craving: a case control study. Hum. Psychopharmacol..

[bib2] Agarwal V., Sitholey P. (2006). A preliminary open trial of olanzapine in paediatric acute and transient psychotic disorders. Indian J. Psychiatr..

[bib3] Alonso-Pedrero L., Bes-Rastrollo M., Marti A. (2019). Effects of antidepressant and antipsychotic use on weight gain: a systematic review. Obes. Rev..

[bib4] Aman M.G., Arnold L.E., McDougle C.J., Vitiello B., Scahill L., Davies M., McCracken J.T., Tierney E., Nash P.L., Posey D.J., Chuang S., Martin A., Shah B., Gonzalez N.M., Swiezy N.B., Ritz L., Koenig K., McGough J., Ghuman J.K., Lindsay R.L. (2005). Acute and long-term safety and tolerability of risperidone in children with autism. J. Child Adolesc. Psychopharmacol..

[bib5] Aman M.G., Rettiganti M., Nagaraja H.N., Hollway J.A., McCracken J., McDougle C.J., Tierney E., Scahill L., Arnold L.E., Hellings J., Posey D.J., Swiezy N.B., Ghuman J., Grados M., Shah B., Vitiello B. (2015). Tolerability, safety, and benefits of risperidone in children and adolescents with autism: 21-month follow-up after 8-week placebo-controlled trial. J. Child Adolesc. Psychopharmacol..

[bib6] Bachmann C.J., Gebhardt S., Lehr D., Haberhausen M., Kaiser C., Otto B., Theisen F.M. (2012). Subjective and biological weight-related parameters in adolescents and young adults with schizophrenia spectrum disorder under clozapine or olanzapine treatment. Z. Kinder JugenPsychiatr. Psychother..

[bib7] Ballon J.S., Pajvani U.B., Mayer L.E.S., Freyberg Z., Freyberg R., Contreras I., Rosenbaum M., Leibel R.L., Lieberman J.A. (2018). Pathophysiology of drug induced weight and metabolic effects: findings from an RCT in healthy volunteers treated with olanzapine, iloperidone, or placebo. J. Psychopharmacol..

[bib8] Barton B.B., Segger F., Fischer K., Obermeier M., Musil R. (2020). Update on weight-gain caused by antipsychotics: a systematic review and meta-analysis. Expet Opin. Drug Saf..

[bib9] Basgul S.S. (2014). Aripiprazole use in children and adolescents: a public hospital child psychiatry outpatient department's experience. Klinik Psikofarmakoloji Bülteni-Bulletin of Clinical Psychopharmacology.

[bib10] Bellisle F. (2009). [Assessing various aspects of the motivation to eat that can affect food intake and body weight control]. Encephale.

[bib11] Bitter I., Treuer T., Dilbaz N., Oyffe I., Ciorabai E.M., Gonzalez S.L., Ruschel S., Salburg J., Dyachkova Y. (2010). Patients' preference for olanzapine orodispersible tablet compared with conventional oral tablet in a multinational, randomized, crossover study. World J. Biol. Psychiatr..

[bib12] Black D.W., Zanarini M.C., Romine A., Shaw M., Allen J., Schulz S.C. (2014). Comparison of low and moderate dosages of extended-release quetiapine in borderline personality disorder: a randomized, double-blind, placebo-controlled trial. Am. J. Psychiatr..

[bib13] Blouin M., Tremblay A., Jalbert M.E., Venables H., Bouchard R.H., Roy M.A., Alméras N. (2008). Adiposity and eating behaviors in patients under second generation antipsychotics. Obesity.

[bib14] Blundell J.E., Gibbons C., Caudwell P., Finlayson G., Hopkins M. (2015). Appetite control and energy balance: impact of exercise. Obes. Rev..

[bib15] Bobo W.V., Epstein R.A., Shelton R.C. (2011). Effects of orally disintegrating vs regular olanzapine tablets on body weight, eating behavior, glycemic and lipid indices, and gastrointestinal hormones: a randomized, open comparison in outpatients with bipolar depression. Ann. Clin. Psychiatr..

[bib16] Boon-Yasidhi V., Jearnarongrit P., Tulayapichitchock P., Tarugsa J. (2014). Adverse effects of risperidone in children with autism spectrum disorders in a naturalistic clinical setting at siriraj hospital, Thailand. Psychiatry J.

[bib17] Calarge C.A., Nicol G., Xie D., Zimmerman B. (2012). Correlates of weight gain during long-term risperidone treatment in children and adolescents. Child Adolesc. Psychiatr. Ment. Health.

[bib18] Calarge C.A., Ziegler E.E. (2013). Iron deficiency in pediatric patients in long-term risperidone treatment. J. Child Adolesc. Psychopharmacol..

[bib19] Cepaityte D., Siafis S., Papazisis G. (2021). Safety of antipsychotic drugs: a systematic review of disproportionality analysis studies. Behav. Brain Res..

[bib20] Chukir T., Mandel L., Tchang B.G., Al-Mulla N.A., Igel L.I., Kumar R.B., Waitman J., Aronne L.J., Shukla A.P. (2021). Metformin-induced weight loss in patients with or without type 2 diabetes/prediabetes: a retrospective cohort study. Obes. Res. Clin. Pract..

[bib21] Cicala G., Barbieri M.A., Santoro V., Tata C., Colucci P.V., Vanadia F., Drago F., Russo C., Cutroneo P.M., Gagliano A., Spina E., Germano E. (2020). Safety and tolerability of antipsychotic drugs in pediatric patients: data from a 1-year naturalistic study. Front. Psychiatr..

[bib22] Cohen J. (2013).

[bib23] Compton M.T., Bakeman R., Capulong L., Pauselli L., Alolayan Y., Crisafio A., King K., Reed T., Broussard B., Shim R. (2020). Associations between two domains of social adversity and recovery among persons with serious mental illnesses being treated in community mental health centers. Community Ment. Health J..

[bib24] Correll C.U., Howes O.D. (2021). Treatment-resistant schizophrenia: definition, predictors, and therapy options. J. Clin. Psychiatr..

[bib25] Coskun M., Zoroglu S.S., Ozturk M. (2011). Risperidone treatment in preschool children with disruptive behavior disorders: a chart review study. Klinik Psikofarmakoloji Bülteni-Bulletin of Clinical Psychopharmacology.

[bib26] Costa e Silva J.A., Alvarez N., Mazzotti G., Gattaz W.F., Ospina J., Larach V., Starkstein S., Oliva D., Cousins L., Tohen M., Taylor C.C., Wang J., Tran P.V. (2001). Olanzapine as alternative therapy for patients with haloperidol-induced extrapyramidal symptoms: results of a multicenter, collaborative trial in Latin America. J. Clin. Psychopharmacol..

[bib27] Daurignac E., Leonard K.E., Dubovsky S.L. (2015). Increased lean body mass as an early indicator of olanzapine-induced weight gain in healthy men. Int. Clin. Psychopharmacol..

[bib28] Davies M., Færch L., Jeppesen O.K., Pakseresht A., Pedersen S.D., Perreault L., Rosenstock J., Shimomura I., Viljoen A., Wadden T.A., Lingvay I. (2021). Semaglutide 2·4 mg once a week in adults with overweight or obesity, and type 2 diabetes (STEP 2): a randomised, double-blind, double-dummy, placebo-controlled, phase 3 trial. Lancet.

[bib29] Davis B.J., Lysaker P.H., Salyers M.P., Minor K.S. (2020). The insight paradox in schizophrenia: a meta-analysis of the relationship between clinical insight and quality of life. Schizophr. Res..

[bib30] Dayabandara M., Hanwella R., Ratnatunga S., Seneviratne S., Suraweera C., de Silva V.A. (2017). Antipsychotic-associated weight gain: management strategies and impact on treatment adherence. Neuropsychiatric Dis. Treat..

[bib31] de Beaurepaire R. (2021). Binge eating disorders in antipsychotic-treated patients with schizophrenia: prevalence, antipsychotic specificities, and changes over time. J. Clin. Psychopharmacol..

[bib32] de Maio D. (1972). Clozapine, a novel major tranquilizer. Clinical experiences and pharmacotherapeutic hypotheses. Arzneimittelforschung.

[bib33] Dell'Osso B., Arici C., Dobrea C., Benatti B., Altamura A.C. (2012). Efficacy, tolerability, compliance, and quality of life of patients with mood disorders switched from quetiapine immediate release to extended release. Int. Clin. Psychopharmacol..

[bib34] Demirkaya S.K., Aksu H., Özgür B.G. (2017). A retrospective study of long acting risperidone use to support treatment adherence in youth with conduct disorder. Clin Psychopharmacol Neurosci.

[bib35] Deng C. (2013). Effects of antipsychotic medications on appetite, weight, and insulin resistance. Endocrinol Metab. Clin. N. Am..

[bib36] DerSimonian R., Laird N. (1986). Meta-analysis in clinical trials. Contr. Clin. Trials.

[bib37] Duval S., Tweedie R. (2000). A nonparametric “trim and fill” method of accounting for publication bias in meta-analysis. J. Am. Stat. Assoc..

[bib38] Findling R.L., Landbloom R.L., Szegedi A., Koppenhaver J., Braat S., Zhu Q., Mackle M., Chang K., Mathews M. (2015). Asenapine for the acute treatment of pediatric manic or mixed episode of bipolar I disorder. J. Am. Acad. Child Adolesc. Psychiatry.

[bib39] Findling R.L., McNamara N.K., Youngstrom E.A., Branicky L.A., Demeter C.A., Schulz S.C. (2003). A prospective, open-label trial of olanzapine in adolescents with schizophrenia. J. Am. Acad. Child Adolesc. Psychiatry.

[bib40] Findling R.L., McNamara N.K., Youngstrom E.A., Stansbrey R.J., Frazier T.W., Lingler J., Otto B.D., Demeter C.A., Rowles B.M., Calabrese J.R. (2011). An open-label study of aripiprazole in children with a bipolar disorder. J. Child Adolesc. Psychopharmacol..

[bib41] Fountaine R.J., Taylor A.E., Mancuso J.P., Greenway F.L., Byerley L.O., Smith S.R., Most M.M., Fryburg D.A. (2010). Increased food intake and energy expenditure following administration of olanzapine to healthy men. Obesity.

[bib42] Gagliano A., Germanò E., Pustorino G., Impallomeni C., D'Arrigo C., Calamoneri F., Spina E. (2004). Risperidone treatment of children with autistic disorder: effectiveness, tolerability, and pharmacokinetic implications. J. Child Adolesc. Psychopharmacol..

[bib43] Garfinkel P.E., Garner D.M., Kaplan A.S., Rodin G., Kennedy S. (1983). Differential diagnosis of emotional disorders that cause weight loss. Can. Med. Assoc. J..

[bib44] Garriga M., Mallorquí A., Serrano L., Ríos J., Salamero M., Parellada E., Gómez-Ramiro M., Oliveira C., Amoretti S., Vieta E., Bernardo M., García-Rizo C. (2019). Food craving and consumption evolution in patients starting treatment with clozapine. Psychopharmacology (Berl).

[bib45] Gebhardt S., Haberhausen M., Krieg J.C., Remschmidt H., Heinzel-Gutenbrunner M., Hebebrand J., Theisen F.M. (2007). Clozapine/olanzapine-induced recurrence or deterioration of binge eating-related eating disorders. J. Neural. Transm..

[bib46] Ghaeli P., Nikvarz N., Alaghband-Rad J., Alimadadi A., Tehrani-Doost M. (2014). Effects of risperidone on core symptoms of autistic disorder based on childhood autism rating scale: an open label study. Indian J. Psychol. Med..

[bib47] Ghanizadeh A. (2016). Twice-weekly aripiprazole for treating children and adolescents with tic disorder, a randomized controlled clinical trial. Ann. Gen. Psychiatr..

[bib48] Ghanizadeh A., Haghighi A. (2014). Aripiprazole versus risperidone for treating children and adolescents with tic disorder: a randomized double blind clinical trial. Child Psychiatr. Hum. Dev..

[bib49] Goluza I., Borchard J., Kiarie E., Mullan J., Pai N. (2017). Exploration of food addiction in people living with schizophrenia. Asian J Psychiatr.

[bib50] Gothelf D., Falk B., Singer P., Kairi M., Phillip M., Zigel L., Poraz I., Frishman S., Constantini N., Zalsman G., Weizman A., Apter A. (2002). Weight gain associated with increased food intake and low habitual activity levels in male adolescent schizophrenic inpatients treated with olanzapine. Am. J. Psychiatr..

[bib51] Guardia J., Segura L., Gonzalvo B., Iglesias L., Roncero C., Cardús M., Casas M. (2004). A double-blind, placebo-controlled study of olanzapine in the treatment of alcohol-dependence disorder. Alcohol Clin. Exp. Res..

[bib52] Han R., Bian Q., Chen H. (2022). Effectiveness of olanzapine in the treatment of anorexia nervosa: a systematic review and meta-analysis. Brain Behav.

[bib53] Hasan A., Bandelow B., Yatham L.N., Berk M., Falkai P., Möller H.J., Kasper S. (2019). WFSBP guidelines on how to grade treatment evidence for clinical guideline development. World J. Biol. Psychiatr..

[bib54] Hellings J.A., Zarcone J.R., Reese R.M., Valdovinos M.G., Marquis J.G., Fleming K.K., Schroeder S.R. (2006). A crossover study of risperidone in children, adolescents and adults with mental retardation. J. Autism Dev. Disord..

[bib55] Henderson D.C., Borba C.P., Daley T.B., Boxill R., Nguyen D.D., Culhane M.A., Louie P., Cather C., Evins A.E., Freudenreich O., Taber S.M., Goff D.C. (2006). Dietary intake profile of patients with schizophrenia. Ann. Clin. Psychiatr..

[bib56] Himmerich H., Minkwitz J., Kirkby K.C. (2015). Weight gain and metabolic changes during treatment with antipsychotics and antidepressants. Endocr., Metab. Immune Disord.: Drug Targets.

[bib57] Ho C.S., Chiu N.C., Tseng C.F., Huang Y.L. (2014). Clinical effectiveness of aripiprazole in short-term treatment of tic disorder in children and adolescents: a naturalistic study. Pediatr Neonatol.

[bib58] Holt R.I.G., Gossage-Worrall R., Hind D., Bradburn M.J., McCrone P., Morris T., Edwardson C., Barnard K., Carey M.E., Davies M.J., Dickens C.M., Doherty Y., Etherington A., French P., Gaughran F., Greenwood K.E., Kalidindi S., Khunti K., Laugharne R., Pendlebury J., Rathod S., Saxon D., Shiers D., Siddiqi N., Swaby E.A., Waller G., Wright S. (2019). Structured lifestyle education for people with schizophrenia, schizoaffective disorder and first-episode psychosis (STEPWISE): randomised controlled trial. Br. J. Psychiatry.

[bib59] Hsiao H.C., Chao H.C., Wang J.J. (2013). Features of problematic eating behaviors among community-dwelling older adults with dementia: family caregivers' experience. Geriatr. Nurs..

[bib60] Ishitobi M., Hiratani M., Kosaka H., Takahashi T., Mizuno T., Asano M., Murata T., Tomoda A., Wada Y. (2012). Switching to aripiprazole in subjects with pervasive developmental disorders showing tolerability issues with risperidone. Prog. Neuro-Psychopharmacol. Biol. Psychiatry.

[bib61] Jackson V.M., Breen D.M., Fortin J.P., Liou A., Kuzmiski J.B., Loomis A.K., Rives M.L., Shah B., Carpino P.A. (2015). Latest approaches for the treatment of obesity. Expet Opin. Drug Discov..

[bib62] Jakobsen A.S., Speyer H., Nørgaard H.C.B., Hjorthoj C., Krogh J., Mors O., Nordentoft M. (2018). Associations between clinical and psychosocial factors and metabolic and cardiovascular risk factors in overweight patients with schizophrenia spectrum disorders - baseline and two-years findings from the CHANGE trial. Schizophr. Res..

[bib63] Jakobsen A.S., Speyer H., Nørgaard H.C.B., Karlsen M., Hjorthøj C., Krogh J., Mors O., Nordentoft M., Toft U. (2018). Dietary patterns and physical activity in people with schizophrenia and increased waist circumference. Schizophr. Res..

[bib64] Kane J.M., Marder S.R., Schooler N.R., Wirshing W.C., Umbricht D., Baker R.W., Wirshing D.A., Safferman A., Ganguli R., McMeniman M., Borenstein M. (2001). Clozapine and haloperidol in moderately refractory schizophrenia: a 6-month randomized and double-blind comparison. Arch. Gen. Psychiatr..

[bib65] Karagianis J., Grossman L., Landry J., Reed V.A., de Haan L., Maguire G.A., Hoffmann V.P., Milev R. (2009). A randomized controlled trial of the effect of sublingual orally disintegrating olanzapine versus oral olanzapine on body mass index: the PLATYPUS Study. Schizophr. Res..

[bib66] Kemner C., Willemsen-Swinkels S.H.N., de Jonge M., Tuynman-Qua H., van Engeland H. (2002). Open-label study of olanzapine in children with pervasive developmental disorder. J. Clin. Psychopharmacol..

[bib67] Kent J.M., Hough D., Singh J., Karcher K., Pandina G. (2013). An open-label extension study of the safety and efficacy of risperidone in children and adolescents with autistic disorder. J. Child Adolesc. Psychopharmacol..

[bib68] Kent J.M., Kushner S., Ning X., Karcher K., Ness S., Aman M., Singh J., Hough D. (2013). Risperidone dosing in children and adolescents with autistic disorder: a double-blind, placebo-controlled study. J. Autism Dev. Disord..

[bib69] Khazaal Y., Chatton A., Claeys F., Ribordy F., Khan R., Zullino D. (2009). Hunger and negative alliesthesia to aspartame and sucrose in patients treated with antipsychotic drugs and controls. Eat. Weight Disord..

[bib70] Khosravi M. (2020). Biopsychosocial factors associated with disordered eating behaviors in schizophrenia. Ann. Gen. Psychiatr..

[bib71] Kim S.F., Huang A.S., Snowman A.M., Teuscher C., Snyder S.H. (2007). From the Cover: antipsychotic drug-induced weight gain mediated by histamine H1 receptor-linked activation of hypothalamic AMP-kinase. Proc. Natl. Acad. Sci. U. S. A..

[bib72] Kinon B.J., Basson B.R., Gilmore J.A., Tollefson G.D. (2001). Long-term olanzapine treatment: weight change and weight-related health factors in schizophrenia. J. Clin. Psychiatr..

[bib73] Kinon B.J., Kaiser C.J., Ahmed S., Rotelli M.D., Kollack-Walker S. (2005). Association between early and rapid weight gain and change in weight over one year of olanzapine therapy in patients with schizophrenia and related disorders. J. Clin. Psychopharmacol..

[bib74] Kluge M., Schuld A., Himmerich H., Dalal M., Schacht A., Wehmeier P.M., Hinze-Selch D., Kraus T., Dittmann R.W., Pollmaecher T. (2007). Clozapine and olanzapine are associated with food craving and binge eating - results from a randomized double-blind study. J. Clin. Psychopharmacol..

[bib75] Kobayashi N., Takano M. (2018). Aripiprazole-induced sleep-related eating disorder: a case report. J. Med. Case Rep..

[bib76] Kraepelin E. (1904).

[bib77] Kroeze W.K., Hufeisen S.J., Popadak B.A., Renock S.M., Steinberg S., Ernsberger P., Jayathilake K., Meltzer H.Y., Roth B.L. (2003). H1-histamine receptor affinity predicts short-term weight gain for typical and atypical antipsychotic drugs. Neuropsychopharmacology.

[bib78] Kryspin-Exner W. (1947). Beiträge zum Verlauf des Körpergewichtes bei Psychosen. Wien Klin. Wochenschr..

[bib79] Küçükerdönmez Ö., Urhan M., Altın M., Hacıraifoğlu Ö., Yıldız B. (2019). Assessment of the relationship between food addiction and nutritional status in schizophrenic patients. Nutr. Neurosci..

[bib80] Kurpad S.S., George S.A., Srinivasan K. (2010). Binge eating and other eating behaviors among patients on treatment for psychoses in India. Eat. Weight Disord..

[bib81] Lappin J.M., Wijaya M., Watkins A., Morell R., Teasdale S., Lederman O., Rosenbaum S., Dick S., Ward P., Curtis J. (2018). Cardio-metabolic risk and its management in a cohort of clozapine-treated outpatients. Schizophr. Res..

[bib82] Larsen J.R., Vedtofte L., Jakobsen M.S.L., Jespersen H.R., Jakobsen M.I., Svensson C.K., Koyuncu K., Schjerning O., Oturai P.S., Kjaer A., Nielsen J., Holst J.J., Ekstrøm C.T., Correll C.U., Vilsbøll T., Fink-Jensen A. (2017). Effect of liraglutide treatment on prediabetes and overweight or obesity in clozapine- or olanzapine-treated patients with schizophrenia spectrum disorder: a randomized clinical trial. JAMA Psychiatr..

[bib83] Lee S.E., Lee N.Y., Kim S.H., Kim K.A., Kim Y.S. (2021). Effect of liraglutide 3.0mg treatment on weight reduction in obese antipsychotic-treated patients. Psychiatr. Res..

[bib84] Lieberman J.A. (2004). Dopamine partial agonists: a new class of antipsychotic. CNS Drugs.

[bib85] Lindsay R.L., Eugene Arnold L., Aman M.G., Vitiello B., Posey D.J., McDougle C.J., Scahill L., Pachler M., McCracken J.T., Tierney E., Bozzolo D. (2006). Dietary status and impact of risperidone on nutritional balance in children with autism: a pilot study. J. Intellect. Dev. Disabil..

[bib86] Litten R.Z., Fertig J.B., Falk D.E., Ryan M.L., Mattson M.E., Collins J.F., Murtaugh C., Ciraulo D., Green A.I., Johnson B., Pettinati H., Swift R., Afshar M., Brunette M.F., Tiouririne N.A.D., Kampman K., Stout R., Grp N.S. (2012). A double-blind, placebo-controlled trial to assess the efficacy of quetiapine fumarate XR in very heavy-drinking alcohol-dependent patients. Alcohol Clin. Exp. Res..

[bib87] Lundgren J.D., Allison K.C., Crow S., O'Reardon J.P., Berg K.C., Galbraith J., Martino N.S., Stunkard A.J. (2006). Prevalence of the night eating syndrome in a psychiatric population. Am. J. Psychiatr..

[bib88] Lyon G.J., Samar S., Jummani R., Hirsch S., Spirgel A., Goldman R., Coffey B.J. (2009). Aripiprazole in children and adolescents with Tourette's disorder: an open-label safety and tolerability study. J. Child Adolesc. Psychopharmacol..

[bib89] Maayan L., Vakhrusheva J., Correll C.U. (2010). Effectiveness of medications used to attenuate antipsychotic-related weight gain and metabolic abnormalities: a systematic review and meta-analysis. Neuropsychopharmacology.

[bib90] Malone R.P., Maislin G., Choudhury M.S., Gifford C., Delaney M.A. (2002). Risperidone treatment in children and adolescents with autism: short- and long-term safety and effectiveness. J. Am. Acad. Child Adolesc. Psychiatry.

[bib91] Mann T., Tomiyama A.J., Westling E., Lew A.M., Samuels B., Chatman J. (2007). Medicare's search for effective obesity treatments: diets are not the answer. Am. Psychol..

[bib92] Masi G., Pisano S., Pfanner C., Milone A., Manfredi A. (2013). Quetiapine monotherapy in adolescents with bipolar disorder comorbid with conduct disorder. J. Child Adolesc. Psychopharmacol..

[bib93] Mathews J., Newcomer J.W., Mathews J.R., Fales C.L., Pierce K.J., Akers B.K., Marcu I., Barch D.M. (2012). Neural correlates of weight gain with olanzapine. Arch. Gen. Psychiatr..

[bib94] McCracken J.T., McGough J., Shah B., Cronin P., Hong D., Aman M.G., Arnold L.E., Lindsay R., Nash P., Hollway J., McDougle C.J., Posey D., Swiezy N., Kohn A., Scahill L., Martin A., Koenig K., Volkmar F., Carroll D., Lancor A., Tierney E., Ghuman J., Gonzalez N.M., Grados M., Vitiello B., Ritz L., Davies M., Robinson J., McMahon D. (2002). Risperidone in children with autism and serious behavioral problems. N. Engl. J. Med..

[bib95] Milaneschi Y., Lamers F., Peyrot W.J., Baune B.T., Breen G., Dehghan A., Forstner A.J., Grabe H.J., Homuth G., Kan C., Lewis C., Mullins N., Nauck M., Pistis G., Preisig M., Rivera M., Rietschel M., Streit F., Strohmaier J., Teumer A., Van der Auwera S., Wray N.R., Boomsma D.I., Penninx B. (2017). Genetic association of major depression with atypical features and obesity-related immunometabolic dysregulations. JAMA Psychiatr..

[bib96] Moe A.A., Scott J.G., Burne T.H., Eyles D.W. (2016). Neural changes induced by antipsychotic administration in adolescence: a review of studies in laboratory rodents. J. Psychopharmacol..

[bib97] Moola S., Munn Z., Tufanaru C., Aromataris E., Sears K., Sfetcu R., Currie M., Qureshi R., Mattis P., Lisy K., Mu P.-F. (2020).

[bib98] Moore J.K., Watson H.J., Harper E., McCormack J., Nguyen T. (2013). Psychotropic drug prescribing in an Australian specialist child and adolescent eating disorder service: a retrospective study. J Eat Disord.

[bib99] Moore N.A., Tye N.C., Axton M.S., Risius F.C. (1992). The behavioral pharmacology of olanzapine, a novel "atypical" antipsychotic agent. J. Pharmacol. Exp. Therapeut..

[bib100] Morell R., Curtis J., Watkins A., Poole J., Fibbins H., Rossimel E., Gerrard M., White A., Teasdale S., Ward P.B., Lappin J. (2019). Cardio-metabolic risk in individuals prescribed long-acting injectable antipsychotic medication. Psychiatr. Res..

[bib101] Murashita M., Kusumi I., Inoue T., Takahashi Y., Hosoda H., Kangawa K., Koyama T. (2005). Olanzapine increases plasma ghrelin level in patients with schizophrenia. Psychoneuroendocrinology.

[bib102] Nagaraj R., Singhi P., Malhi P. (2006). Risperidone in children with autism: randomized, placebo-controlled, double-blind study. J. Child Neurol..

[bib103] Navari R.M., Pywell C.M., Le-Rademacher J.G., White P., Dodge A.B., Albany C., Loprinzi C.L. (2020). Olanzapine for the treatment of advanced cancer-related chronic nausea and/or vomiting: a randomized pilot trial. JAMA Oncol..

[bib104] Ngai Y.F., Devlin A.M., Panagiotopoulos C. (2018). Risperidone but not quetiapine treatment is associated with increased appetite but not satiety hormones in children during an oral glucose tolerance test: a pilot study. J. Clin. Psychopharmacol..

[bib105] NICE, N.C.C.f.M.H. (2014).

[bib106] Nuntamool N., Ngamsamut N., Vanwong N., Puangpetch A., Chamnanphon M., Hongkaew Y., Limsila P., Suthisisang C., Wilffert B., Sukasem C. (2017). Pharmacogenomics and efficacy of risperidone long-term treatment in Thai autistic children and adolescents. Basic Clin. Pharmacol. Toxicol..

[bib107] Okamoto H., Shono K., Nozaki-Taguchi N. (2019). Low-dose of olanzapine has ameliorating effects on cancer-related anorexia. Cancer Manag. Res..

[bib108] Onor M.L., Saina M., Trevisiol M., Cristante T., Aguglia E. (2007). Clinical experience with risperidone in the treatment of behavioral and psychological symptoms of dementia. Prog. Neuro-Psychopharmacol. Biol. Psychiatry.

[bib109] Ouzzani M., Hammady H., Fedorowicz Z., Elmagarmid A. (2016).

[bib110] Page M.J., McKenzie J.E., Bossuyt P.M., Boutron I., Hoffmann T.C., Mulrow C.D., Shamseer L., Tetzlaff J.M., Akl E.A., Brennan S.E., Chou R., Glanville J., Grimshaw J.M., Hróbjartsson A., Lalu M.M., Li T., Loder E.W., Mayo-Wilson E., McDonald S., McGuinness L.A., Stewart L.A., Thomas J., Tricco A.C., Welch V.A., Whiting P., Moher D. (2021). The PRISMA 2020 statement: an updated guideline for reporting systematic reviews. Bmj.

[bib111] Park S., Yi K.K., Kim M.-S., Hong J.P. (2013). Effects of ziprasidone and olanzapine on body composition and metabolic parameters: an open-label comparative pilot study. Behav. Brain Funct..

[bib112] Peled O., Lavan O., Stein J., Vinograd I., Yahel A., Valevski A., Weizman A., Kimmel-Tamir E., Apter A., Fennig S., Yaniv I., Bernfeld Y., Benaroya-Milshtein N. (2020). Psychopharmacology in the pediatric oncology and bone marrow transplant units: antipsychotic medications palliate symptoms in children with cancer. J. Child Adolesc. Psychopharmacol..

[bib113] Pillay J., Boylan K., Newton A., Hartling L., Vandermeer B., Nuspl M., MacGregor T., Featherstone R., Carrey N. (2018). Harms of antipsychotics in children and young adults: a systematic review update. Can. J. Psychiatr..

[bib114] Piparva K.G., Buch J.G., Chandrani K.V. (2011). Analysis of adverse drug reactions of atypical antipsychotic drugs in psychiatry OPD. Indian J. Psychol. Med..

[bib115] Platzer M., Fellendorf F.T., Bengesser S.A., Birner A., Dalkner N., Hamm C., Lenger M., Maget A., Pilz R., Queissner R., Reininghaus B., Reiter A., Mangge H., Zelzer S., Kapfhammer H.-P., Reininghaus E.Z. (2021). The relationship between food craving, appetite-related hormones and clinical parameters in bipolar disorder. Nutrients.

[bib116] Platzer M., Fellendorf F.T., Bengesser S.A., Birner A., Dalkner N., Hamm C., Lenger M., Maget A., Pilz R., Queissner R., Reininghaus B., Reiter A., Mangge H., Zelzer S., Kapfhammer H.P., Reininghaus E.Z. (2020). The relationship between food craving, appetite-related hormones and clinical parameters in bipolar disorder. Nutrients.

[bib117] Poyurovsky M., Fuchs C., Pashinian A., Levi A., Faragian S., Maayan R., Gil-Ad I. (2007). Attenuating effect of reboxetine on appetite and weight gain in olanzapine-treated schizophrenia patients: a double-blind placebo-controlled study. Psychopharmacology (Berl).

[bib118] Poyurovsky M., Isaacs I., Fuchs C., Schneidman M., Faragian S., Weizman R., Weizman A. (2003). Attenuation of olanzapine-induced weight gain with reboxetine in patients with schizophrenia: a double-blind, placebo-controlled study. Am. J. Psychiatr..

[bib119] Pozzi M., Bertella S., Cattaneo D., Molteni M., Perrone V., Carnovale C., Antoniazzi S., Clementi E., Radice S. (2013). Are non-serious adverse reactions to psychiatric drugs really non-serious?. J. Child Adolesc. Psychopharmacol..

[bib120] Qurashi I., Stephenson P., Chu S., Duffy C., Husain N., Chaudhry I. (2015). An evaluation of subjective experiences, effects and overall satisfaction with clozapine treatment in a UK forensic service. THERAPEUTIC ADVANCES IN PSYCHOPHARMACOLOGY.

[bib121] Razjouyan K., Danesh A., Khademi M., Davari-Ashtiani R., Noorbakhsh S. (2018). A comparative study of risperidone and aripiprazole in attention deficit hyperactivity disorder in children under six years old: a randomized double-blind study. Iran. J. Pediatr. (Engl. Ed.).

[bib122] Ribeiro E.L.A., de Mendonça Lima T., Vieira M.E.B., Storpirtis S., Aguiar P.M. (2018). Efficacy and safety of aripiprazole for the treatment of schizophrenia: an overview of systematic reviews. Eur. J. Clin. Pharmacol..

[bib123] Roerig J.L., Mitchell J.E., de Zwaan M., Crosby R.D., Gosnell B.A., Steffen K.J., Wonderlich S.A. (2005). A comparison of the effects of olanzapine and risperidone versus placebo on eating behaviors. J. Clin. Psychopharmacol..

[bib124] Roerig J.L., Steffen K.J., Mitchell J.E. (2011). Atypical antipsychotic-induced weight gain: insights into mechanisms of action. CNS Drugs.

[bib125] Rognoni C., Bertolani A., Jommi C. (2021). Second-generation antipsychotic drugs for patients with schizophrenia: systematic literature review and meta-analysis of metabolic and cardiovascular side effects. Clin. Drug Invest..

[bib126] Rubino D., Abrahamsson N., Davies M., Hesse D., Greenway F.L., Jensen C., Lingvay I., Mosenzon O., Rosenstock J., Rubio M.A., Rudofsky G., Tadayon S., Wadden T.A., Dicker D. (2021). Effect of continued weekly subcutaneous semaglutide vs placebo on weight loss maintenance in adults with overweight or obesity: the STEP 4 randomized clinical trial. JAMA.

[bib127] Sankaranarayanan A., Johnson K., Mammen S.J., Wilding H.E., Vasani D., Murali V., Mitchison D., Castle D.J., Hay P. (2021). Disordered eating among people with schizophrenia spectrum disorders: a systematic review. Nutrients.

[bib128] Scahill L., Jeon S., Boorin S.J., McDougle C.J., Aman M.G., Dziura J., McCracken J.T., Caprio S., Arnold L.E., Nicol G., Deng Y., Challa S.A., Vitiello B. (2016). Weight gain and metabolic consequences of risperidone in young children with autism spectrum disorder. J. Am. Acad. Child Adolesc. Psychiatry.

[bib129] Schmid C.H., Lau J., McIntosh M.W., Cappelleri J.C. (1998). An empirical study of the effect of the control rate as a predictor of treatment efficacy in meta-analysis of clinical trials. Stat. Med..

[bib130] Seifarth C., Schehler B., Schneider H.J. (2013). Effectiveness of metformin on weight loss in non-diabetic individuals with obesity. Exp. Clin. Endocrinol. Diabetes.

[bib131] Sentissi O., Viala A., Bourdel M.C., Kaminski F., Bellisle F., Olié J.P., Poirier M.F. (2009). Impact of antipsychotic treatments on the motivation to eat: preliminary results in 153 schizophrenic patients. Int. Clin. Psychopharmacol..

[bib132] Sharma E., Venkatasubramanian G., Varambally S., Sivakumar P.T., Subbakrishna D.K., Gangadhar B.N. (2014). Antipsychotic induced metabolic changes & treatment response: a prospective study. Asian J Psychiatr.

[bib133] Shi Q., Wang Y., Hao Q., Vandvik P.O., Guyatt G., Li J., Chen Z., Xu S., Shen Y., Ge L., Sun F., Li L., Yu J., Nong K., Zou X., Zhu S., Wang C., Zhang S., Qiao Z., Jian Z., Li Y., Zhang X., Chen K., Qu F., Wu Y., He Y., Tian H., Li S. (2022). Pharmacotherapy for adults with overweight and obesity: a systematic review and network meta-analysis of randomised controlled trials. Lancet.

[bib134] Siskind D.J., Lee M., Ravindran A., Zhang Q., Ma E., Motamarri B., Kisely S. (2018). Augmentation strategies for clozapine refractory schizophrenia: a systematic review and meta-analysis. Aust. N. Z. J. Psychiatr..

[bib135] Smith R.C., Rachakonda S., Dwivedi S., Davis J.M. (2012). Olanzapine and risperidone effects on appetite and ghrelin in chronic schizophrenic patients. Psychiatr. Res..

[bib136] Snyder R., Turgay A., Aman M., Binder C., Fisman S., Carroll A. (2002). Effects of risperidone on conduct and disruptive behavior disorders in children with subaverage IQs. J. Am. Acad. Child Adolesc. Psychiatry.

[bib137] Srivastava S., Wang P.W., Hill S.J., Childers M.E., Keller K.L., Ketter T.A. (2012). Pilot study of the efficacy of double-blind, placebo-controlled one-week olanzapine stabilization therapy in heterogeneous symptomatic bipolar disorder patients. J. Psychiatr. Res..

[bib138] Stip E., Lungu O.V., Anselmo K., Letourneau G., Mendrek A., Stip B., Lipp O., Lalonde P., Bentaleb L.A. (2012). Neural changes associated with appetite information processing in schizophrenic patients after 16 weeks of olanzapine treatment. Transl. Psychiatry.

[bib139] Taylor V.H., Stonehocker B., Steele M., Sharma A.M. (2012). An overview of treatments for obesity in a population with mental illness. Can. J. Psychiatr..

[bib140] Teasdale S.B., Ward P.B., Jarman R., Wade T., Rossimel E., Curtis J., Lappin J., Watkins A., Samaras K. (2018). Is obesity in young people with psychosis a foregone conclusion? Markedly excessive energy intake is evident soon after antipsychotic initiation. Front. Psychiatr..

[bib141] Teff K.L., Rickels K., Alshehabi E., Rickels M.R. (2015). Metabolic impairments precede changes in hunger and food intake following short-term administration of second-generation antipsychotics. J. Clin. Psychopharmacol..

[bib142] Teff K.L., Rickels M.R., Grudziak J., Fuller C., Nguyen H.L., Rickels K. (2013). Antipsychotic-induced insulin resistance and postprandial hormonal dysregulation independent of weight gain or psychiatric disease. Diabetes.

[bib143] Theisen F.M., Linden A., König I.R., Martin M., Remschmidt H., Hebebrand J. (2003). Spectrum of binge eating symptomatology in patients treated with clozapine and olanzapine. J. Neural. Transm..

[bib144] Tohen M., Baker R.W., Altshuler L.L., Zarate C.A., Suppes T., Ketter T.A., Milton D.R., Risser R., Gilmore J.A., Breier A., Tollefson G.A. (2002). Olanzapine versus divalproex in the treatment of acute mania. Am. J. Psychiatr..

[bib145] Tohen M., Ketter T.A., Zarate C.A., Suppes T., Frye M., Altshuler L., Zajecka J., Schuh L.M., Risser R.C., Brown E., Baker R.W. (2003). Olanzapine versus divalproex sodium for the treatment of acute mania and maintenance of remission: a 47-week study. Am. J. Psychiatr..

[bib146] Tollefson G.D., Beasley C.M., Tran P.V., Street J.S., Krueger J.A., Tamura R.N., Graffeo K.A., Thieme M.E. (1997). Olanzapine versus haloperidol in the treatment of schizophrenia and schizoaffective and schizophreniform disorders: results of an international collaborative trial. Am. J. Psychiatr..

[bib147] Treuer T., Hoffmann V.P., Chen A.K.-P., Irimia V., Ocampo M., Wang G., Singh P., Holt S. (2009). Factors associated with weight gain during olanzapine treatment in patients with schizophrenia or bipolar disorder: results from a six-month prospective, multinational, observational study. World J. Biol. Psychiatr..

[bib148] Troost P.W., Lahuis B.E., Steenhuis M.P., Ketelaars C.E.J., Buitelaar J.K., Van Engeland H., Scahill L., Minderaa R.B., Hoekstra P.J. (2005). Long-term effects of risperidone in children with autism spectrum disorders: a placebo discontinuation study. J. Am. Acad. Child Adolesc. Psychiatry.

[bib149] Wadden T.A., Bailey T.S., Billings L.K., Davies M., Frias J.P., Koroleva A., Lingvay I., O'Neil P.M., Rubino D.M., Skovgaard D., Wallenstein S.O.R., Garvey W.T. (2021). Effect of subcutaneous semaglutide vs placebo as an adjunct to intensive behavioral therapy on body weight in adults with overweight or obesity: the STEP 3 randomized clinical trial. JAMA.

[bib150] Wang L., Du M., Wang K., Khandpur N., Rossato S.L., Drouin-Chartier J.-P., Steele E.M., Giovannucci E., Song M., Zhang F.F. (2022). Association of ultra-processed food consumption with colorectal cancer risk among men and women: results from three prospective US cohort studies. BMJ.

[bib151] Weizman S., Shelef A., Bloemhof Bris E., Stryjer R. (2021). A double-blind, placebo-controlled trial of bupropion add-on to olanzapine or risperidone in overweight individuals with schizophrenia. J. Clin. Psychopharmacol..

[bib152] Wen Chi L., Lin S.C., Chang S.H., Wu H.S. (2015). Factors associated with hyperphagic behavior in patients with dementia living at home. Biol. Res. Nurs..

[bib153] Wilding J.P.H., Batterham R.L., Calanna S., Davies M., Van Gaal L.F., Lingvay I., McGowan B.M., Rosenstock J., Tran M.T.D., Wadden T.A., Wharton S., Yokote K., Zeuthen N., Kushner R.F. (2021). Once-Weekly semaglutide in adults with overweight or obesity. N. Engl. J. Med..

[bib154] Williams R., Smith M., Wright D. (2021). Anorexia: a literature review of young people's experiences of hospital treatment. Nurs. Child. Young People.

[bib155] Yektaş Ç., Tufan A.E. (2018). Prescribing trends of atypical antipsychotic drugs in an outpatient unit of a child and adolescent clinic in Turkey. Clin. Neuropharmacol..

[bib156] Zhao Y.J., Lin L., Teng M., Khoo A.L., Soh L.B., Furukawa T.A., Baldessarini R.J., Lim B.P., Sim K. (2016). Long-term antipsychotic treatment in schizophrenia: systematic review and network meta-analysis of randomised controlled trials. BJPsych Open.

