# Supplementary material for: Eating cognitions, emotions and behaviour under treatment with second generation antipsychotics: A systematic review and meta-analysis
Source: J Psychiatr Res. 2023 Apr;160:137–62. doi: 10.1016/j.jpsychires.2023.02.006 (PMC10682412; doi:10.1016/j.jpsychires.2023.02.006)
Supplement: Multimedia component 2 [file mmc2.docx]

**S.2. PRISMA 2020 abstract checklist**

| Title |  |  |  |
| --- | --- | --- | --- |
| **Title** | 1 | Identify the report as a systematic review. | Yes |
| **Background** |  |  |  |
| **Objectives** | 2 | Provide an explicit statement of the main objective(s) or question(s) the review addresses. | Yes |
| **Methods** |  |  |  |
| **Eligibility Criteria** | 3 | Specify the inclusion and exclusion criteria for the review. | Yes |
| **Information Sources** | 4 | Specify the information sources (e.g. databases, registers) used to identify studies and the date when each was last searched. | Yes |
| **Risk Of Bias** | 5 | Specify the methods used to assess risk of bias in the included studies. | Yes |
| **Synthesis Of Results** | 6 | Specify the methods used to present and synthesize results. | Yes |
| **Results** |  |  |  |
| **Included Studies** | 7 | Give the total number of included studies and participants and summarise relevant characteristics of studies. | Yes |
| **Synthesis Of Results** | 8 | Present results for main outcomes, preferably indicating the number of included studies and participants for each. If meta-analysis was done, report the summary estimate and confidence/credible interval. If comparing groups, indicate the direction of the effect (i.e. which group is favoured). | Yes |
| **Discussion** |  |  |  |
| **Limitations Of Evidence** | 9 | Provide a brief summary of the limitations of the evidence included in the review (e.g. study risk of bias, inconsistency and imprecision). | Yes |
| **Interpretation** | 10 | Provide a general interpretation of the results and important implications. | Yes |
| **Other** |  |  |  |
| **Funding** | 11 | Specify the primary source of funding for the review. | Yes |
| **Registration** | 12 | Provide the register name and registration number. | Yes |
