# Supplementary material for: Eating cognitions, emotions and behaviour under treatment with second generation antipsychotics: A systematic review and meta-analysis
Source: J Psychiatr Res. 2023 Apr;160:137–62. doi: 10.1016/j.jpsychires.2023.02.006 (PMC10682412; doi:10.1016/j.jpsychires.2023.02.006)
Supplement: Multimedia component 3 [file mmc3.docx]

S.3. Search strategy

| Database | Search Query | |
| --- | --- | --- |
| PubMed | #1 | ("eating behavior" OR "binge eating" OR "food craving" OR "carbohydrate craving" OR "food intake" OR "dietary intake" OR "food preference" OR “appetite” OR “hunger” OR “food addiction” OR “junk food” OR “food choice” OR “eating habits” OR “motivation to eat” OR “food responsiveness” OR “overeating” OR “hyperphagia” OR “energy intake” OR “caloric intake” OR “desire to eat” OR “Polyphagia” OR “pica”). |
|  | #2 | (“antipsychotic” OR “amisulpride” OR “aripiprazole” OR “asenapine” OR “clozapine” OR “lurasidone” OR “olanzapine” OR “paliperidone” OR “quetiapine” OR “risperidone” OR “cariprazine” OR “brexpiprazole” OR “clotiapine” OR “iloperidone” OR “molindone” OR “ziprasidone”). |
|  | #3 | #1 AND #2. |
| Web of Science (all databases) | #1 | **AB=("eating behavior" OR “binge eating" OR "food craving" OR "carbohydrate craving" OR "food intake" OR "dietary intake" OR "food preference" OR “appetite” OR “hunger” OR “food addiction” OR “junk food” OR “food choice” OR “eating habits” OR “motivation to eat” OR “food responsiveness”** OR “overeating” OR “hyperphagia” OR “energy intake” OR “caloric intake” OR “desire to eat” OR “Polyphagia” OR “pica”**).** |
|  | #2 | **AB=(“antipsychotic” OR “amisulpride” OR “aripiprazole” OR “asenapine” OR “clozapine” OR “lurasidone” OR “olanzapine” OR “paliperidone” OR “quetiapine” OR “risperidone” OR “cariprazine” OR “brexpiprazole” OR “clotiapine” OR “iloperidone” OR “molindone" OR “ziprasidone”).** |
|  | #3 | **#1 AND #2.** |
| APA PsycInfo (Ovid) | #1 | Eating behavior or binge eating or food craving or carbohydrate craving or food intake or dietary intake or food preference or appetite or hunger or food addiction or junk food or food or food choice or eating habits or motivation to eat or food responsiveness or overeating or hyperphagia or energy intake or caloric intake or desire to eat or Polyphagia or pica (keyword). |
|  | #2 | antipsychotic or amisulpride or aripiprazole or asenapine or clozapine or lurasidone or olanzapine or paliperidone or quetiapine or risperidone or cariprazine or brexpiprazole or clotiapine or iloperidone or molindone or ziprasidone (keyword). |
|  | #3 | **Select #1 and #2, combine with “AND”.** |
