# Supplementary material for: Eating cognitions, emotions and behaviour under treatment with second generation antipsychotics: A systematic review and meta-analysis
Source: J Psychiatr Res. 2023 Apr;160:137–62. doi: 10.1016/j.jpsychires.2023.02.006 (PMC10682412; doi:10.1016/j.jpsychires.2023.02.006)
Supplement: Multimedia component 4 [file mmc4.docx]

**S.4. Inclusion and exclusion criteria**

| **Inclusion criteria** | **Exclusion criteria** |
| --- | --- |
| *Study design* | |
| Original articles, prospective and retrospective. | Case reports, review articles, perspective papers, letters (without data), master or doctoral theses, meta-analyses & animal studies. |
| *Outcomes* | |
| Eating behaviour, food intake, regulation of appetite & food preferences. | Weight change or hormonal changes. |
| *Study participants* | |
| Humans of all diagnoses including psychiatric disorders, Male and female with all ages. | Animal studies. |
| *Intervention* | |
| Studies measuring outcomes of SGAs treatment | Studies measuring the effect of other medications and/or the combination of treatments to overcome weight gain caused by SGAs use and/or combination of treatments where the outcome of other drug is measured together with the SGAs. |
| *Language* | |
| Only English language publications. | All other languages |
| *Accessibility* | |
| Access to full published text and methodology. | All articles with no full text and methodology available even if abstracts were accessible. |
| *Setting* | |
| Inpatients, outpatients and healthy volunteers | N/A |
| *Quality assessment* | |
| Less than 50 % responses to “no” or “unclear” (low risk of bias) | More than 50 % of responses to “no” or “unclear” (high risk of bias) |
