# Supplementary material for: Eating cognitions, emotions and behaviour under treatment with second generation antipsychotics: A systematic review and meta-analysis
Source: J Psychiatr Res. 2023 Apr;160:137–62. doi: 10.1016/j.jpsychires.2023.02.006 (PMC10682412; doi:10.1016/j.jpsychires.2023.02.006)
Supplement: Multimedia component 5 [file mmc5.docx]

**S.5. Summary of quality assessment of included studies**

| a. JBI cross-sectional critical appraisal tool (N=25) |
| --- |

| Publication | Were the criteria for inclusion in the sample clearly defined? | Were the study subjects and the setting described in detail? | Was the exposure measured in a valid and reliable way? | Were objective, standard criteria used for measurement of the condition? | Were confounding factors identified? | Were strategies to deal with confounding factors stated? | Were the outcomes measured in a valid and reliable way? | Was appropriate statistical analysis used? | Decision | Comment |
| --- | --- | --- | --- | --- | --- | --- | --- | --- | --- | --- |
| Bachmann et al. (2012) | + | + | N/A | + | - | - | + | + | Include |  |
| Blouin et al. (2008) | + | + | + | + | + | + | + | + | Include |  |
| Boon-Yasidhi et al. (2014) | + | + | + | + | - | - | - | U/C | Include | - Newly developed questionnaire - Results of statistical analyses of interest were not reported |
| Calarge et al. (2012) | + | + | + | + | + | + | + | + | Include |  |
| Calarge and Ziegler (2013) | + | + | + | + | + | + | + | + | Include |  |
| de Beaurepaire (2021) | + | + | + | + | U/C | U/C | + | + | Include |  |
| Gebhardt et al. (2007) | + | + | + | + | U/C | U/C | + | + | Include |  |
| Goluza et al. (2017) | - | + | + | + | + | + | + | + | Include | - Diagnosis not stated - Undetailed inclusion criteria |
| Henderson et al. (2006) | - | - | N/A | + | - | - | + | + | Include |  |
| Jakobsen et al. (2018b) | + | + | + | + | - | - | + | + | Include |  |
| Khazaal et al. (2009) | + | + | + | + | + | + | + | + | Include |  |
| Kurpad et al. (2010) | + | + | + | + | U/C | U/C | + | + | Include |  |
| Lappin et al. (2018) | U/C | - | + | + | - | - | + | + | Include | - Non-validated questionnaire - Results of statistical analysis of interest not reported |
| Lundgren et al. (2006) | - | + | + | + | U/C | U/C | + | + | Include | - Undetailed inclusion criteria |
| Morell et al. (2019) | + | + | + | + | - | - | + | + | Include |  |
| Ngai et al. (2018) | + | + | + | + | - | - | + | + | Include |  |
| Nuntamool et al. (2017) | + | + | + | + | - | - | + | + | Include |  |
| Peled et al. (2020) | + | + | + | + | - | - | + | + | Include |  |
| Platzer et al. (2020) | + | + | + | + | + | U/C | + | + | Include |  |
| Pozzi et al. (2013) | + | + | + | + | + | + | + | + | Include |  |
| Qurashi et al. (2015) | + | + | + | + | U/C | U/C | + | - | Include | - Missing statistical analysis for outcomes of interest. |
| Sentissi et al. (2009) | + | + | + | + | U/C | U/C | + | + | Include |  |
| Teasdale et al. (2018) | + | + | + | + | - | - | + | + | Include |  |
| Theisen et al. (2003) | - | + | + | + | - | - | + | + | Include | - Undetailed inclusion and exclusion criteria. |
| Yektaş and Tufan (2018) | + | + | + | + | + | - | + | + | Include |  |

| 1. JBI case-control critical appraisal tool (N=1) |
| --- |

| Publication | Were the groups comparable other than the presence of disease in cases or the absence of disease in controls? | Were cases and controls matched appropriately? | Were the same criteria used for identification of cases and controls? | Was exposure measured in a standard, valid and reliable way? | Was exposure measured in the same way for cases and controls? | Were confounding factors identified? | Were strategies to deal with confounding factors stated? | Were outcomes assessed in a standard, valid and reliable way for cases and controls? | Was the exposure period of interest long enough to be meaningful? | Was appropriate statistical analysis used? | Decision | Comment |
| --- | --- | --- | --- | --- | --- | --- | --- | --- | --- | --- | --- | --- |
| Abbas and Liddle (2013) | + | + | + | + | + | - | N/A | + | + | + | Include | - Changes in food craving after medication used was not measured due to the nature of study. |

| 1. JBI randomized controlled trials critical appraisal tool (N=29) |
| --- |

| Publication | Was true randomization used for assignment of participants to treatment groups? | Was allocation to treatment groups concealed? | Were treatment groups similar at the baseline? | Were participants blind to treatment assignment? | Were those delivering treatment blind to treatment assignment? | Were outcomes assessors blind to treatment assignment? | Were treatment groups treated identically other than the intervention of interest? | Was follow up complete and if not, were difference between groups in terms of their follow up adequately described and analysed? | Were participants analysed in the groups to which they were randomized? | Were outcomes measured in the same way for treatment groups? | Were outcomes measured in a reliable way? | Was appropriate statistical analysis used? | Was the trial design appropriate, and any deviations from the standard RCT design (individual randomization, parallel groups) accounted for in the conduct and analysis of the trial? | Decision | Comment |
| --- | --- | --- | --- | --- | --- | --- | --- | --- | --- | --- | --- | --- | --- | --- | --- |
| Aman et al. (2005) | + | U/C | + | + | + | + | + | + | + | + | + | + | + | Include |  |
| Ballon et al. (2018) | + | U/C | + | + | + | U/C | + | + | + | + | + | + | + | Include |  |
| Black et al. (2014) | + | + | + | + | + | + | + | + | + | + | + | + | + | Include |  |
| Daurignac et al. (2015) | + | + | + | + | + | U/C | + | + | + | + | + | + | + | Include |  |
| Findling et al. (2015) | + | + | + | + | + | + | + | + | + | + | + | + | + | Include |  |
| Fountaine et al. (2010) | + | + | U/C | + | + | U/C | + | + | + | + | + | + | + | Include |  |
| Ghanizadeh (2016) | + | + | + | U/C | U/C | U/C | + | + | + | + | + | + | + | Include |  |
| Guardia et al. (2004) | + | + | + | + | + | U/C | + | + | + | + | + | + | + | Include |  |
| Hellings et al. (2006) | + | U/C | U/C | + | - | U/C | + | + | + | + | + | + | + | Include |  |
| Kane et al. (2001) | + | + | + | + | + | U/C | + | + | + | + | + | + | + | Include |  |
| Karagianis et al. (2009) | + | U/C | + | + | + | + | + | + | + | + | + | + | + | Include |  |
| Kent et al. (2013b) | + | + | + | + | + | + | + | + | + | + | + | + | + | Include |  |
| Lindsay et al. (2006) | + | U/C | + | + | + | + | + | + | + | + | + | - | + | Include | - Missing statistical analysis for outcomes of interest. |
| Litten et al. (2012) | + | + | + | + | + | U/C | + | + | + | + | + | + | + | Include |  |
| McCracken et al. (2002) | + | + | + | + | U/C | + | + | + | + | + | + | + | + | Include |  |
| Nagaraj et al. (2006) | + | + | + | + | + | + | + | + | + | + | + | + | + | Include |  |
| Navari et al. (2020) | + | + | + | + | + | + | + | + | + | + | + | + | + | Include |  |
| Razjouyan et al. (2018) | + | + | + | + | + | + | + | + | + | + | + | + | + | Include |  |
| Roerig et al. (2005) | + | + | + | + | + | + | + | + | + | + | + | + | + | Include |  |
| Snyder et al. (2002) | + | + | + | + | + | U/C | + | + | + | + | + | + | + | Include |  |
| Srivastava et al. (2012) | + | + | + | + | + | U/C | + | + | + | + | + | + | + | Include |  |
| Teff et al. (2013) | + | + | + | + | + | + | + | + | + | + | + | + | + | Include |  |
| Tohen et al. (2002) | + | + | + | + | + | - | + | + | + | + | + | + | + | Include |  |
| Tohen et al. (2003) | + | + | + | + | + | U/C | + | + | + | + | + | + | + | Include |  |
| Tollefson et al. (1997) | + | U/C | + | + | + | U/C | + | + | + | + | + | + | + | Include |  |
| Ghanizadeh and Haghighi (2014) | + | + | U/C | + | + | + | + | + | + | + | + | + | + | Include |  |
| Jakobsen et al. (2018a) | + | U/C | + | - | - | + | + | + | + | + | + | + | + | Include |  |
| Scahill et al. (2016) | + | + | + | U/C | + | + | + | + | + | + | + | + | + | Include |  |
| Smith et al. (2012) | + | - | + | - | - | - | + | + | + | + | + | + | + | Include |  |

| 1. JBI cohort critical appraisal tool (N=8) |
| --- |

| Publication | Were the two groups similar and recruited from the same population? | Were the exposures measured similarly to assign people to both exposed and unexposed groups? | Was the exposure measured in a valid and reliable way? | Were confounding factors identified? | Were strategies to deal with confounding factors stated? | Were the groups / participants free of the outcome at the start of the study (or at the moment of exposure)? | Were the outcomes measured in a valid and reliable way? | Was the follow up time reported and sufficient to be long enough for outcomes to occur? | Was follow up complete, and if not, were the reasons to loss to follow up described and explored? | Were strategies to address incomplete follow up utilized? | Was appropriate statistical analysis used? | Decision | comment |
| --- | --- | --- | --- | --- | --- | --- | --- | --- | --- | --- | --- | --- | --- |
| Aman et al. (2015) | + | + | + | + | + | + | + | + | + | N/A | + | Include |  |
| Cicala et al. (2020) | N/A | N/A | + | U/C | U/C | N/A | + | + | + | N/A | + | Include |  |
| Garriga et al. (2019) | N/A | N/A | + | + | + | - | + | + | + | N/A | + | Include |  |
| Teff et al. (2015) | + | + | + | - | - | + | + | + | + | N/A | + | Include |  |
| Piparva et al. (2011) | N/A | + | + | - | - | - | - | + | - | + | + | Include |  |
| Murashita et al. (2005) | N/A | N/A | + | U/C | U/C | + | + | + | + | N/A | + | Include |  |
| Treuer et al. (2009) | N/A | N/A | + | + | - | - | + | + | + | + | + | Include |  |
| Stip et al. (2012) | N/A | N/A | + | - | - | + | + | + | + | N/A | + | Include |  |

| 1. JBI quasi experimental critical appraisal tool (N=29) |
| --- |

| Publication | Is it clear in the study what is the ‘cause’ and what is the ‘effect’ (i.e., there is no confusion about which variable comes first)? | Were the participants included in any comparisons similar? | Were the participants included in any comparisons receiving similar treatment/care, other than the exposure or intervention of interest? | Was there a control group? | Were there multiple measurements of the outcome both pre and post the intervention/exposure? | Was follow up complete and if not, were differences between groups in terms of their follow up adequately described and analyzed? | Were the outcomes of participants included in any comparisons measured in the same way? | Were outcomes measured in a reliable way? | Was appropriate statistical analysis used? | Decision | Comment |
| --- | --- | --- | --- | --- | --- | --- | --- | --- | --- | --- | --- |
| Agarwal and Sitholey (2006) | + | - | + | - | + | + | + | + | + | Include |  |
| Bitter et al. (2010) | + | + | + | - | + | + | + | + | + | Include |  |
| Bobo et al. (2011) | + | + | + | - | + | + | + | + | + | Include |  |
| Costa e Silva et al. (2001) | + | - | - | - | + | + | - | + | + | Include |  |
| Findling et al. (2003) | + | + | - | - | + | + | + | + | + | Include |  |
| Findling et al. (2011) | + | + | + | - | + | + | + | + | + | Include |  |
| Gagliano et al. (2004) | + | - | - | - | + | + | - | + | + | Include |  |
| Ghaeli et al. (2014) | + | + | - | - | + | + | + | + | + | Include |  |
| Gothelf et al. (2002) | + | + | - | - | + | + | + | + | + | Include |  |
| Ho et al. (2014) | + | + | - | - | - | + | - | + | + | Include |  |
| Ishitobi et al. (2012) | + | - | - | - | N/A | N/A | - | + | + | Include |  |
| Kemner et al. (2002) | + | - | + | - | + | + | + | + | + | Include |  |
| Kent et al. (2013a) | + | + | + | + | + | + | + | + | + | Include |  |
| Lyon et al. (2009) | + | - | - | - | + | + | - | + | + | Include |  |
| Malone et al. (2002) | + | - | - | - | + | + | + | + | + | Include |  |
| Park et al. (2013) | + | + | + | - | + | + | + | + | + | Include |  |
| Troost et al. (2005) | + | + | + | + | + | + | + | + | + | Include |  |
| Mathews et al. (2012) | + | + | + | - | + | + | + | + | + | Include |  |
| Onor et al. (2007) | + | - | + | - | + | + | + | + | + | Include |  |
| Kinon et al. (2005) | + | + | + | - | + | + | + | + | + | Include |  |
| Kluge et al. (2007) | + | + | + | - | + | + | + | + | + | Include |  |
| Dell'Osso et al. (2012) | + | + | - | - | + | + | + | + | + | Include |  |
| Masi et al. (2013) | + | - | - | - | + | + | - | + | + | Include |  |
| Sharma et al. (2014) | + | - | - | - | + | + | - | + | + | Include |  |
| Basgul (2014) | + | - | - | - | - | + | - | + | + | Include | - Missing statistical analysis for outcomes of interest |
| Coskun et al. (2011) | + | - | - | - | + | + | - | + | + | Include |  |
| Demirkaya et al. (2017) | + | - | - | - | + | + | - | + | + | Include |  |
| Moore et al. (2013) | + | - | - | - | - | N/A | + | + | + | Include |  |
| Okamoto et al. (2019) | + | - | - | - | + | + | - | + | + | Include | - Small sample size and limited follow-up period. |

+: Yes; -: No; N/A: Not applicable; U/C: Unclear. Randomised trials were also assessed using the randomized controlled trials JBI critical appraisal tool. Quasi experimental quality assessment tool was used for open-label, non-randomised trials, data from RCTs, prospective and retrospective studies.
