# Supplementary material for: Eating cognitions, emotions and behaviour under treatment with second generation antipsychotics: A systematic review and meta-analysis
Source: J Psychiatr Res. 2023 Apr;160:137–62. doi: 10.1016/j.jpsychires.2023.02.006 (PMC10682412; doi:10.1016/j.jpsychires.2023.02.006)
Supplement: Multimedia component 6 [file mmc6.docx]

**S.6. Qualitative appraisal of additional studies reporting eating-related outcomes**

**Appetite and hunger**

Besides studies included in the meta-analyses and odds ratio calculations, and studies included in the visual demonstration of participants reporting appetite increase with SGAs, other studies included in this review showed conflicting results. Of these, seven studies reported that SGAs did not affect hunger ratings (Bachmann et al., 2012; Bobo et al., 2011; Mathews et al., 2012; Roerig et al., 2005; Stip et al., 2012; Teff et al., 2015; Teff et al., 2013). Even though some of these studies reported numerical but not statistical increase in hunger ratings (Roerig et al., 2005) and others showed gender-related differences (Bachmann et al., 2012).

Treatment with SGAs did not alter the hunger ratings in (Bachmann et al., 2012; Bobo et al., 2011; Mathews et al., 2012; Stip et al., 2012). Only one study reported a decrease in hunger ratings throughout the follow-up periods specifically after the consumption of sweet stimuli such as aspartame and sucrose (Khazaal et al., 2009). Decrease in appetite was reported in a small number of participants treated with SGAs (Agarwal and Sitholey, 2006; Coskun et al., 2011; Ghanizadeh and Haghighi, 2014; Ho et al., 2014; Kent et al., 2013a; McCracken et al., 2002; Piparva et al., 2011; Snyder et al., 2002; Tollefson et al., 1997).

**Food craving**

Five publications reported food craving as an outcome (Abbas and Liddle, 2013; Bobo et al., 2011; Garriga et al., 2019; Kluge et al., 2007; Platzer et al., 2020). Two of these concluded that there was no change seen in food cravings in all follow-up points and between different study arms (Bobo et al., 2011; Garriga et al., 2019). Interestingly, two studies concluded that participants treated with SGAs had higher craving rates for sweets (Kluge et al., 2007; Platzer et al., 2020) and fatty foods (Kluge et al., 2007) while another concluded that craving scores were higher in FGA treated when compared to olanzapine treated participants (Abbas and Liddle (2013).

**Dietary disinhibition, binge eating and loss of control over eating**

Besides studies included in the meta-analyses, others seen no change in disinhibited eating before and after treatment (Bachmann et al., 2012; Bobo et al., 2011; Stip et al., 2012) except for Mathews et al. (2012) which showed that disinhibited eating subscale of the TFEQ was higher one week after the treatment with olanzapine.

Within studies identified in this review, only a small number reported results relating to eating disorders diagnoses and symptomologies with SGAs use. Six studies investigated the correlation between SGAs treatment with binge eating and BED (de Beaurepaire, 2021; Gebhardt et al., 2007; Kluge et al., 2007; Kurpad et al., 2010; Moore et al., 2013; Theisen et al., 2003). A higher number of participants in the olanzapine group screened positive to binge eating when compared to clozapine treated participants (Theisen et al., 2003). Similarly, Kluge et al. (2007) found that binge eating was seen earlier in the olanzapine group and is numerically higher when compared to the clozapine group. However, risperidone users showed higher binge eating symptomology when compared to the olanzapine treated participants (Kurpad et al., 2010). Moore et al. (2013) reported that SGAs use was associated with new onset of binge eating and that four cases had clinical concerns that olanzapine treatment is accused to aggravate their BE. A higher percentage of participant treated with olanzapine met the diagnostic criteria for BED when compared to clozapine (Theisen et al., 2003) and a higher percentage of participants partially fulfilled the BED criteria (de Beaurepaire, 2021). The correlation between BED and SGAs use declines in the longer term of more than 2 years. Besides other studies reporting that olanzapine induces binge eating (Kluge et al., 2007; Theisen et al., 2003). It was also shown that exposure to olanzapine and clozapine may induce relapse in binge eating in patients with previous ED diagnosis Gebhardt and colleagues (Gebhardt et al., 2007).

Only two studies reported the effect of SGAs on BN and numbers of bulimic episodes (de Beaurepaire, 2021; Theisen et al., 2003). When compared to clozapine treated participants, twice of participants treated with olanzapine met the diagnostic criteria for BN in (Theisen et al., 2003) while none met diagnostic criteria for BN in (de Beaurepaire, 2021).

NE was seen in 30 % of (de Beaurepaire, 2021)’s sample and was more prevalent in the clozapine and olanzapine treated groups. Also, SGAs use was seen more frequently in participants who were diagnosed with night eating syndrome (Lundgren et al., 2006).

**Dietary restraints**

Studies investigated dietary restraint found no correlation between SGAs exposure and the dietary restraint subscale of the TFEQ (Bachmann et al., 2012; Bobo et al., 2011; Mathews et al., 2012; Stip et al., 2012).

**Dietary composition, quality and eating habits**

Within the studies which measured the eating behaviour of participants treated with SGAs, some reported that participants consumed less fruits and vegetables (Henderson et al., 2006; Jakobsen et al., 2018b; Lappin et al., 2018), wholegrain carbohydrate choices (Henderson et al., 2006; Lappin et al., 2018), meats (Henderson et al., 2006), dairy products (Henderson et al., 2006; Lappin et al., 2018), healthy fat containing food choices (Lappin et al., 2018) while they consumed higher amount of calories from sweets or added sugars (Henderson et al., 2006; Jakobsen et al., 2018b; Lappin et al., 2018; Morell et al., 2019), savoury discretionary food choices (Lappin et al., 2018; Morell et al., 2019) and sweet drinks (Lappin et al., 2018; Morell et al., 2019).
